# *PtiCYP85A3*, a BR C-6 Oxidase Gene, Plays a Critical Role in Brassinosteroid-Mediated Tension Wood Formation in Poplar

**DOI:** 10.3389/fpls.2020.00468

**Published:** 2020-04-24

**Authors:** Yanli Jin, Chunyan Yu, Chunmei Jiang, Xiaotong Guo, Bei Li, Cuiting Wang, Fanjing Kong, Hongxia Zhang, Haihai Wang

**Affiliations:** ^1^College of Agriculture, Ludong University, Yantai, China; ^2^National Key Laboratory of Plant Molecular Genetics, Institute of Plant Physiology and Ecology, Shanghai Institutes for Biological Sciences, Chinese Academy of Sciences, Shanghai, China; ^3^The Key Laboratory of Molecular Module-Based Breeding of High Yield and Abiotic Resistant Plants in the Universities of Shandong, Institute for Advanced Study of Coastal Ecology, Ludong University, Yantai, China; ^4^Hubei Collaborative Innovation Center for Green Transformation of Bio-Resources, College of Life Sciences, Hubei University, Wuhan, China; ^5^Ministry of Natural Resources Key Laboratory of Saline Lake Resources and Environments, Institute of Mineral Resources, Chinese Academy of Geological Sciences, Beijing, China

**Keywords:** brassinosteroids, G-layer, poplar, *PtiCYP85A3*, tension wood, xylem

## Abstract

In angiosperm trees, the gelatinous layer (G-layer) takes a great part of the fiber cell wall in the tension wood (TW). However, the mechanism underlying G-layer formation in poplar is largely unknown. In this work, we demonstrate that G-layer formation in poplar TW cells is regulated by brassinosteroid (BR) and its signaling. *PtiCYP85A3*, a key BR biosynthesis gene, was predominantly expressed in the xylem of TW, accompanied with a relatively higher castasterone (CS) accumulation, than in the xylem of opposite wood (OW). A wider expression zone of BZR1, a key transcriptional factor in BR singling pathway, was also observed in G-fiber cells on TW side than in wood fiber cells on the OW side, as indicated by immunohistochemistry assays. Transgenic poplar plants overexpressing *PtiCYP85A3* produced thicker G-layer with higher cellulose proportion, and accumulated more BZR1 protein in the xylem of TW than did the wild type (WT) plants. Expression of most TW-associated *CesAs*, which were induced by 2, 4-epibrassinolide, an active BR, and inhibited by brassinazole, a BR biosynthesis inhibitor, were also up-regulated in the xylem of TW in transgenic plants compared to that in WT plants. Further studies with dual-luciferase assays demonstrated that the promoters of *PtiCesAs* were activated by PtiMYB128, a TW specific transcription factor, which was then regulated by BZR1. All these results indicate that BR plays a crucial role in the G-layer formation of TW fiber cells by regulating the expression of *BZR1*, *PtiMYB128*, and *PtiCesAs* in poplar.

## Introduction

Tension wood (TW) is a kind of reaction wood formed in angiosperm plants as a response to gravity. When plants are subjected to environmental forces, such as landslide, typhoon, flood, and snow storm, TW is formed on the upper side of leaning stems to generate a strong tensile force which helps to pull the inclined stems back to the vertical position (Okuyama et al., 1994; Yoshida et al., 1999, 2000; Yamamoto et al., 2002; Clair et al., 2006; Ruelle et al., 2006; Coutand et al., 2007; Clair et al., 2010). Tension wood is generally characterized by the presence of gelatinous xylem fiber cells with a thick inner gelatinous cell wall layer (G-layer), which contains a high proportion of cellulose, and a low proportion of lignin, various non-cellulosic polysaccharides and glycosylated proteins (Timell, 1969; Goswami et al., 2008; Déjardin et al., 2010; Gorshkova et al., 2015; Guedes et al., 2017; Felten et al., 2018).

Cellulose, composed of linear chains of β-1,4-linked Glc units, is synthesized by the cellulose synthase complexes (CSCs) consisting of cellulose synthases (CESAs) (Zhang et al., 2018). In Arabidopsis, CESA proteins are categorized into six classes: CESA1, CESA3, and CESA6 (including CESA2, CESA5, CESA6, and CESA9) responsible for primary cell wall cellulose biosynthesis, and CESA4, CESA7, and CESA8 responsible for secondary cell wall cellulose biosynthesis (Taylor et al., 2003; Desprez et al., 2007; Persson et al., 2007). In the poplar genome, a total number of 17 *CESA* genes have been indentified and the nomenclature for *CesAs*, *PtiCesA1-A/B*, *PtiCesA3-A/B/C/D*, *PtiCesA4*, *PtiCesA6-A/B/C/D/E/F*, *PtiCesA7-A/B*, and *PtiCesA8-A/B*, was updated based on the alignments of *Populus CesA* gene family with the Arabidopsis *CesA*s (Kumar et al., 2009). Consistent with the CESAs in Arabidopsis, three classes of CESAs, CESA4, CESA7a/b, and CESA8a/b, are involved in cellulose biosynthesis in secondary wall and G-layer formation.

Previous studies have shown that plant hormones such as auxin, ethylene, and gibberellin (GA) were involved in TW formation (Little and Pharis, 1995; Mellerowicz et al., 2001; Pilate et al., 2004; Kwon, 2008). In poplar, although the balance of endogenous auxin level was not significantly altered, the expression of several *Aux*/*IAA* genes changed during TW formation, indicating that auxin may not directly regulate the production of TW (Moyle et al., 2002; Hellgren et al., 2004). Ethylene has been confirmed as a key regulator in TW formation (Andersson-Gunnerås et al., 2003; Love et al., 2009). A poplar ACC oxidase gene, which was induced by gravitational irritation, displayed an asymmetric expression between TW and OW (Andersson-Gunnerås et al., 2003). Upon treatment with 1-methylcyclopropene (1-MCP), an ethylene perception inhibitor, TW formation was inhibited (Love et al., 2009). On the other hand, application of exogenous ethylene or its precursor 1-aminocyclopropane-1-carboxylic acid (ACC) induced G-layer formation and altered cellulose microfibril angle in absence of gravitational stimulus in aspen (Felten et al., 2018). In addition, many genes related to cell expansion and cell wall modification for gelatinous layer induction were regulated by ethylene signaling (Felten et al., 2018). In a weeping type of *Prunus spachiana*, GA promoted the formation of TW on the upper side of branches, and prevented the bending of its branches (Nakamura et al., 1994; Baba et al., 1995; Yoshida et al., 1999). In several species of angiosperm trees, exogenous application of GA to their vertical stems also induced TW formation in the absence of gravitational stimulus (Funada et al., 2008). In transgenic poplar, GA mediated TW formation via the regulation of fasciclin-like arabinogalactan protein gene expression (Wang et al., 2017).

As one of the six classes of phytohormones, BRs also play a key role in plant growth, reproduction, and response to biotic and abiotic stresses (Kim et al., 2005). In Arabidopsis, BRs are synthesized by a list of enzymes step by step, including deetiolated2 (DET2) (Li et al., 1996), constitutive photomorphogenesis and dwarfism (CPD) (Szekeres et al., 1996), dwarf4 (DWF4) (Choe et al., 1998), BR-6-oxidase1 (BR6ox1) (Bishop et al., 1999), and rotundfolia3 (ROT3) (Kim et al., 2005). To date, more than 70 BRs have been identified from the entire plant kingdom (Saleh et al., 2006). Among them, castasterone (CS) and brassinolide (BL) were shown to be the most important BRs (Altmann, 1999). A cytochrome P450, AtCYP85A2, known as a BR C-6 oxidase, catalyzes the conversion of CS to BL. When the function of AtCYP85A2 was knocked out, the Arabidopsis *cyp85a2-2* mutant produced dark green curled leaves with shortened petioles compared with those of the wild type plants. On the other hand, when *AtCYP85A2* was overexpressed, both vegetative and reproductive growth was enhanced in transgenic Arabidopsis plants (Kim et al., 2005). The functions of BRs in other herbaceous and woody plants such as rice, tomato, grape, and pea were also examined in some details (Mori et al., 2002; Nomura et al., 2005; Symons et al., 2006; Jager et al., 2007). However, the biological functions of *CYP85A2* in TW formation in woody plants are still not fully clarified.

Previously, we reported that overexpression of *PtiCYP85A3*, the homology of *AtCYP85A2*, promoted the endogenous CS content, and enhanced the growth and biomass production in transgenic poplar (Jin et al., 2017). In this work, we examined the possible function of BR in TW formation by manipulating the expression of *PtiCYP85A3* in transgenic poplar plants. We found that, by activating BR signaling, *PtiCYP85A3* positively regulated G-layer formation of TW fiber cells in poplar.

## Materials and Methods

### Plant Materials and Growth Conditions

*Populus trichocarpa* genotype Nisqually-1, a commercial clone Shanxin yang (*P. davidiana Dode* × *P. bolleana Lauche*) and transgenic Shanxin yang overexpressing *PtiCYP85A3* (lines L3, L5, and L8) were used in this study (Jin et al., 2017). Generally, *in vitro*-grown plants were sub-cultured monthly by aseptically transferring shoot apices to fresh MS medium supplemented with 0.1 mg l^–1^ of 1-naphthaleneacetic acid (Murashige and Skoog, 1962). Plantlets were then transferred into individual pots and grown in a greenhouse under a 12 h light/12 h dark photoperiod. The temperature was kept at 21–25°C in daytime and 15–18°C at night. All plants were well watered according to the evaporation demands during different growth stages, and fertilized biweekly with water-soluble fertilizers (Plant-Soul, China). Nisqually-1 was used for gene cloning only. Wild type and transgenic Shanxin yang were used for all the other experiments.

### TW Induction and Histochemical Staining

Before TW induction, 2-month-old poplar plants grown in greenhouse were fixed to sticks to make sure all plants grow upstraight. To induce TW formation, poplar plants were tilted to a 45° angle for 14 days. Over 10 plants from wild type and each transgenic line were used for each treatment, and three replicates were carried out for each treatment. In this way, TW and OW were produced on the upper and under sides of leaning stems, respectively. The middle part of stems under the bended point was used to isolate the xylem tissues (without any pith) of tension wood side (TW-X) and opposite wood side (OW-X). Phloem tissues of tension wood side (TW-P) and opposite wood side (OW-P) in the barks were also isolated.

Tension wood staining was performed as described previously (Wang et al., 2017). Briefly, stem segments were cut from the tilted plant, ∼50 stem sections were fixed with 2% formaldehyde and subsequently passed over a gradient ethanol series. Then the sections were embedded in paraffin, and about 10 μm thick sections were cut out with a rotary microtome. After dewaxing, they were stained with 1% aqueous safranin-O (MP, United States) and subsequently with 1% aqueous astra-blue (Santa Cruz, United States) as described previously (Srebotnik and Messner, 1994). High power images were captured under bright field using an ECLIPSE 80i microscope (Nikon, Japan) and low power images were taken under the SMZ800 microscope (Nikon, Japan).

### BR Content Assays

The xylem tissues of TW or OW (TW-X or OW-X), and the bark of TW or OW (TW-P or OW-P) isolated from WT plants were grounded into fine powder in liquid nitrogen for BR content determination as described previously (Jin et al., 2017). Three replicates were carried out with over 5 g dry material for each sample and each experiment. The variability was indicated with the standard deviation (SD).

### Quantitative Real-Time RT-PCR

For the expression analysis of *PtiCYP85A3*, cellulose synthase genes (*CesAs*), and MYB transcription factor genes (*MYBs*) during TW formation, total RNA was extracted from TW-X, TW-P, OW-X, and OW-P of WT plants using RNAiso Reagent (Takara, Japan). After treated with DNase I (Promega, United States), a total amount of 2 μg total RNA was subjected to reverse transcription using the HiScript^®^ II Q RT SuperMix for qPCR (+gDNA wiper) (Vazyme, China). Quantitative real-time RT PCR (qRT-PCR) was performed using an AceQ qPCR SYBR Green Master Mix (Vazyme Biotech, China) and a CFX Connect Real-Time System (Bio-Rad, United States). Three independent replicates of measurements were performed for each sample.

For the expression analyses of *CesA* genes, TW-X of the middle parts of inclined stems from WT and transgenic plants were used for RNA extraction. The relative expression of each target gene was normalized using the house keeping gene *Pd* × *lEF1*β. Gene specific primers used in this study were designed according to the sequences of *P. trichocarpa* and listed in Supplementary Table S1. For genes cloned from *P. trichocarpa*, prefix *Pti* was used in gene names. For gene investigation in *P. davidiana* x *bolleana*, prefix *Pd* × *l* was used in gene names.

### Exogenous EBL and Brassinazole Treatments

To analyze the responses of *CesA*s and *MYB128* to BR, stem segments of the 3th to 4th internodes of 2-month-old WT (Shangxin yang) plants were cut into 1 mm slices, and at least 60 stem sections from 20 stem segments were soaked in 100 nM EBL for each treatment (10, 30, 60, or 220 min). Then, the stem sections were used for RNA extraction and qRT-PCR analyses.

For analyses of the effects of brassinazole (Brz, one of BR biosynthesis inhibitors) on the expressions of *CesAs* and *MYB128*, wild type Shanxin yang plans were tilted to 45° for 10 days and painted with lanolin containing 5 mM brassinazole (Sigma, United States) once every 2 days. Then, TW-X of the middle part of inclined stems of 20 plants was used for RNA extraction and further qRT-PCR analyses.

### Cellulose Quantification

TW-X separated from the middle parts of 20 inclined stems of WT and each transgenic lines (L3, L5, and L8) were cut into small pieces, ground into fine powder in liquid nitrogen. Then, cell wall material (CWM) was isolated by sequentially washing the samples with 70% (v/v) ethanol, chloroform:methanol (1:1) and acetone as described previously (Foster et al., 2010). Starch was removed from the pellet by incubating the sample in 1 ml of 0.1 M sodium acetate buffer (pH 5.0) with amylase (50 μg ml^–1^, Sigma, United States) and pullulanase (Sigma, United States) at 37°C for 12 h. After washed three times with water, the resultant CWM was suspended with acetone and dried at 35°C for 12 h. Cell wall material was used to determine the contents of cellulose.

To determine the cellulose content, CWM was incubated in 1 ml of Updegraff reagent for 30 min at 100°C, and then washed three times with 1 ml of acetone. The pellet (crystalline cellulose) was completely hydrolyzed into glucose in 175 μl of 72% sulfuric acid at room temperature for 45 min. After the addition of 825 μl water, 10 μl of each sample of the supernatant and 90 ml of water was pipetted into separate cells of 96-well polystyrene microtiter plates before the addition of 200 μl of freshly prepared Anthrone reagent. The plate was heated for 30 min at 80°C, and the absorption at 625 nm was measured after being cooled to room temperature (Foster et al., 2010). Three independent replicates of measurements were performed for each sample.

### Antibody Preparation

For Western blot analyses, OsBZR1 polyclonal antibody was purchased from Beijing Genomics Institute (BGI)^1^. Arabidopsis CesA7 (At5g17420) and CesA8 (At4g18780) polyclonal antibody were bought from Agrisera^2^. Plant β-actin antibody and secondary antibodies were purchased from ABclonal^3^.

### Western Blotting Analyses

For Western blotting analyses, total proteins were extracted from the TW-X tissues of inclined WT and transgenic plants using RIPA buffer consisting of 1 mM PMSF, and separated on 10% SDS-PAGE gel. After electrotransfering of the proteins onto polyvinylidene difluoride membranes, the membranes were blocked with TBST buffer (10 mM Tris/HCl, pH 7.5, 0.1% NaCl, 0.05% Tween 20) supplemented with 5% non-fat dried milk for 1 h. The membranes were incubated with primary antibody (diluted at 1:1000) overnight at 4°C. Afterward, the membranes were rinsed three times with TBST buffer and incubated with the secondary antibodies (peroxidase-labeled anti-rabbit antibodies) at a dilution of 1:5000 for 1 h. After washed three times with TBST buffer (5 min each), the membranes were incubated in LumiGLO for chemiluminescence detection (KPL, United States) and then imaged with a Tanon 5500 electrophoresis system (Tanon, China).

### Immunolocalization of BZR1

Immunohistochemical analyses of BZR1 in TW of WT plants were performed as described previously (Wang et al., 2015). Stem segments of inclined WT plants were fixed overnight in 0.1 M PBS (pH 7.5) containing 4% paraformaldehyde, and embedded in paraffin. The slides were spread with polylysine before the sections were fixed. After deparaffinization and dehydration, the sections were washed twice with PBS buffer. The samples were blocked with 5% bovine serum albumin (BSA) in PBS for 1 h at room temperature. Subsequently, they were incubated with anti-OsBZR1 antibodies (diluted at 1:30 with 0.1 M PBS containing 0.1% BSA) at room temperature for 1 h. In the negative control, BZR1 antibody was omitted. After rinsed three times in PBS, the samples were incubated with FITC secondary antibody (diluted at 1:50 in the same buffer) at room temperature for 1 h. Finally, the samples were rinsed with PBS buffer for three times and mounted with a cover glass for photographing. Images were captured under a confocal microscope Zeiss LSM 510 (META, Germany).

### Transient Transcription Dual-Luciferase Assays

For dual-luciferase assays, the LUC reporter constructs were generated by cloning the promoters of *PtiCesA4*, *PtiCesA7-A*, and *PtiMYB128* into the pGreenII0800-LUC, respectively (Hellens et al., 2005). The CaMV 35S promoter-driven transcriptional factor effector constructs were generated by inserting PtiMYB128 or AtBZR1 into the pGreenII62-SK, respectively (Hellens et al., 2005). To detect the induction of PtiMYB128 to the promoters of *PtiCesAs*, the effector of PtiMYB128 and each LUC reporter construct of *PtiCesAs* were respectively co-expressed in poplar leaf protoplasts. To test the transcription activity of AtBZR1 to the PtiMYB128 promoter, the effector of AtBZR1 was co-transfected with *PtiMYB128* LUC reporter construct in poplar leaf protoplasts. Poplar leaf protoplast extraction and transformation were performed as described previously (Wang et al., 2013). After 16 h, transfected cells were collected and homogenized in 300 μl of passive lysis buffer. The crude extract (20 μl) was mixed with 40 μl of luciferase assay buffer and firefly luciferase (LUC) activity was measured using a GLOMAX 20/20 luminometer (Promega, Wisconsin, United States). Stop and Glow Buffer (40 μl) was then added to the reaction solution and renillia luciferase (REN) activity was measured. LUC/REN ratio was used to represent the relative activity of the transcriptional factors on the driving promoters. Three replicates were carried out for each assay, and the variability was indicated with the standard deviation (SD).

### Statistics

All data were obtained from at least three independent experiments with three biological replicates each. For statistical analyses, Student’s *t*-test was used to generate every *P*-value. All the tests were two-tailed.

## Results

### Castasterone Is Accumulated in the Xylem of TW

**FIGURE 1:**
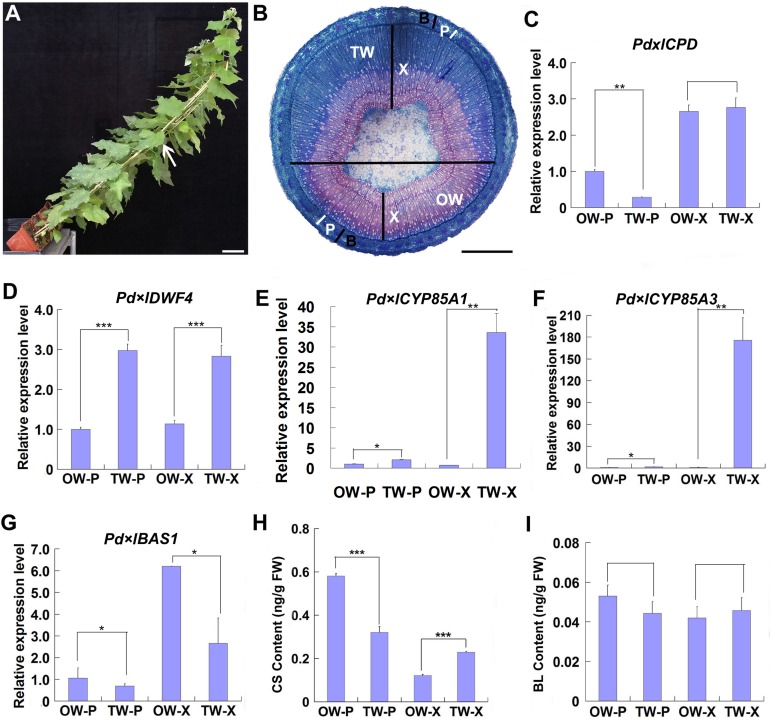
Gene expression and BR content analyses during TW formation. Two-month-old wild type Shanxin yang plants (WT) were fixed to sticks and grown at an angle of 45° in greenhouse to induce TW formation. **(A)** Poplar plants grown in greenhouse for TW induction. Bar = 5 cm. The stem position used for safranin-O and astra-blue staining was indicated with a whit arrow. **(B)** Cross sections from the middle part of the inclined stem were stained with safranin-O and astra-blue. TW was stained into blue color. The horizontal line indicates the division of TW side and OW side. Bar = 1 mm. TW, tension wood; OW, opposite wood; X, xylem tissues; P, phloem tissues; B, bark including phloem tissues. The white bars indicate phloem. **(C–F)** Relative expression of putative BR biosynthesis and metabolic genes in poplar. The expression levels of *Pd* × *lCPD* **(C)**, *Pd* × *lDWF4* **(D)**, *Pd* × *lCYP85A3* **(E)**, and *Pd* × *lBAS1* **(F)** in different tissues of reaction wood were determined. Expression in the phloem tissue of OW was set to 1. Error bars represent the SDs from three biological replicates. **(G,H)** Content analyses of CS **(G)** and BL **(H)** in different tissues of reaction wood. OW-P, phloem tissues on opposite wood side; TW-P, phloem tissues on tension wood side; OW-X, xylem tissues without any pith on opposite wood side; TW-X, xylem tissues without any pith on tension wood side; BL, brassinolide; CS, castasterone. Data were means ± SD (*n* = 9) of three independent biological replicates with three technical replicates each. *, **, and *** indicate significant difference at *P* < 0.05, *P* < 0.01, and *P* < 0.001, respectively (Student’s *t*-test). Gene Potri ID numbers were provided in Supplementary Table S1.

Since TW can be induced by mechanical stress, as done by gravitational stimulation (Jourez et al., 2001; Jourez and Avella-Shaw, 2003; Déjardin et al., 2010), we inclined wild type poplar plants to a 45 degree when grown in greenhouse to induce TW formation (Figure 1A). After 2 weeks, TW was obviously produced on the upper side of leaning stems, and was stained into blue color with safranin/astra blue double staining (Figure 1B).

To explore the possible function of BR in TW formation, we first examined the expression of genes associated with BR biosynthesis and metabolism. Poplar *CPD*, *DWF4*, *CYP85A1*, *CYP85A3*, and *BAS1*, the homologes of Arabidopsis *CPD*, *DWF4*, *CYP85A2*, and *BAS1*, were selected. Although no significant increase was observed in the expression of *Pd* × *lCPD*, increased expression of *Pd* × *lDWF4* in TW, including both xylem and phloem (TW-X and TW-P), was detected, as compared with that in OW (Figures 1C,D). Both *Pd* × *lCYP85A1* and *Pd* × *lCYP85A3* were predominately expressed in the xylem of TW (Figures 1E,F), whereas *Pd* × *lBAS1*, the homologe of Arabidopsis *BAS1*, encoding a cytochrome P450 monooxygenase (CYP734A1, formerly CYP72B1) which inactivates active brassinosteroids (BRs) such as brassinolide (BL) and castasterone (CS), was highly expressed in the xylem of OW (Figure 1G; Turk et al., 2005). The up-regulated expression of BR synthesis genes and the down-regulated expression of BR metabolic genes in TW may have promoted BR accumulation in the xylem of TW. Therefore, we examined the content of BRs in the xylem and phloem of both TW and OW. We found that although no significant difference was detected in the content of BL, asymmetric distribution of CS was observed in the tissues of TW and OW. A higher CS content was detected in the phloem of OW and the xylem of TW (Figures 1H,I), suggesting that CS may be the major BR involved in TW formation.

### BR Signaling Is Enhanced in the Xylem of TW

**FIGURE 2:**
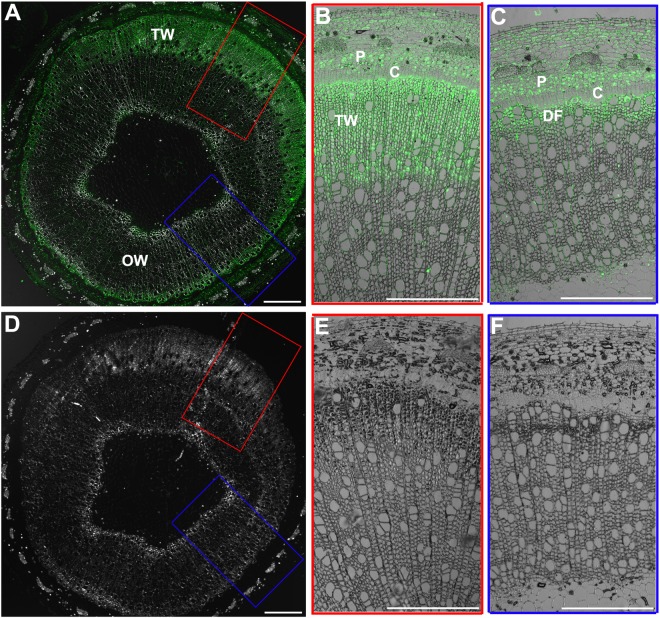
Immunohistochemical analyses of BZR1 protein during TW formation. **(A)** Cross sections of the inclined stem of wild type Shanxin yang plants (WT) were hybridized with anti-OsBZR1 antibodies. The cross lines indicate the division between TW and OW. TW, tension wood; OW, opposite wood. Scale bar = 1 mm. **(B,C)** Higher magnification of the images in **(A)**. Bar = 0.5 mm. **(D)** Cross sections of the inclined stem was hybridized with PBS as control. Scale bar = 1 mm. **(E,F)** Higher magnification of the images in **(D)**. DF, differentiating fibers; DGF, differentiating G-layer fibers. Bar = 0.5 mm.

BZR1/BES1 protein is the key positive transcription factor in the signaling pathway of BRs. The increased CS content in the xylem of TW could be connected with an enhanced BR signaling. To confirm this hypothesis, we investigated the distribution of BZR1 protein during TW formation in WT plants by fluorescence immunohistochemistry test using an OsBZR1 antibody. A very strong BZR1/BES1 protein signal was detected in the G-fiber cells on the TW side (a wide band), but only a weak BZR1/BES1 protein signal was detected in the wood fiber cell on the opposite wood side (a narrow band) (Figures 2A–C). No fluorescence signal was detected in the control section (Figures 2D–F). These results indicate that BR signaling is activated during TW formation.

### *PtiCYP85A3* Promotes G-Layer Formation in the Xylem Fiber Cells of TW

**FIGURE 3:**
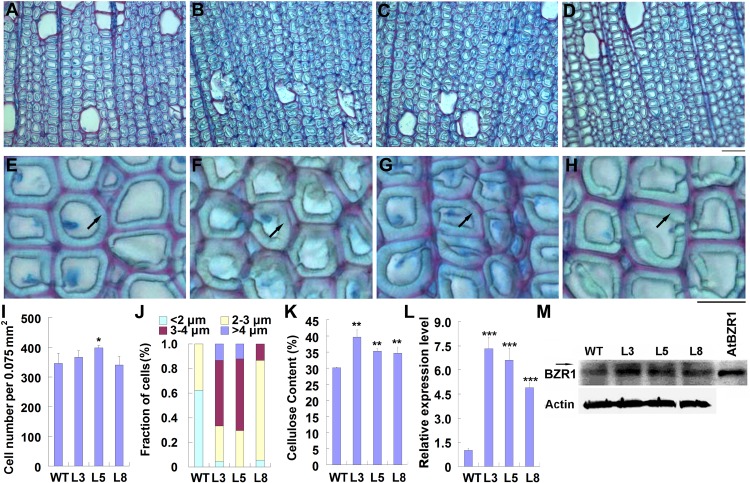
Overexpression of *PtiCYP85A3* prompted gelatinous-layer (G-layer) formation in the TW xylem fiber cells of Shangxin yang plants. **(A–D)** Images to show the gelatinous layer in the TW xylem fiber cells of wild type (WT) **(A)** and transgenic lines L3 **(B)**, L5 **(C)**, and L8 **(D)**. G, G-layer. Bar = 15 μm. **(E–H)** Higher magnification of the images in **(A–D)**, respectively. **(I–K)** Cell number per area **(I)**, fraction of cells with different G-lay thicknesses **(J)**, and cellulose content **(K)** in the TW xylem of WT and different transgenic lines. Data were means ± SD (*n* = 3) of three independent biological replicates. For analyses with different G-lay thicknesses in **(J)**, 300 cells were counted (*n* = 300). **(L)** The expression levels of *PtiCYP85A3* in the TW xylem of WT and different transgenic lines. Data were means ± SD (*n* = 3) of three independent biological replicates. **(M)** Western blotting analyses of BZR1 protein in the TW xylem of WT and different transgenic lines. A total amount of 40 μg proteins extracted from the TW xylem were separated on 10% SDS-PAGE and hybridized with OsBZR1 antibodies (Anti-BZR1) or plant actin antibodies (Anti-Actin). The putative phosphorylated band of BZR1 was indicated with an arrow. M, protein molecular weight marker; WT, wide type Shangxin yang; L3, L5, and L8, different transgenic lines; AtBZR1, proteins from the leaf protoplasts overexpressing *AtBZR1*. *, **, and *** indicate significant difference at *P* < 0.05, *P* < 0.01, and *P* < 0.001, respectively (Student’s *t*-test).

Previously, we reported that overexpression of *PtiCYP85A3* in poplar promoted the accumulation of CS, and significantly improved the growth and biomass of transgenic plants (Jin et al., 2017). To understand the possible effects of BRs on TW formation, we compared the fiber cells in the TW of wild type and transgenic plants by histochemical staining. Although the stem diameter of transgenic plants was greater than that of WT plants, the ratio of TW to OW in the xylem area was not significantly changed in transgenic plants (Supplementary Figures S1A,B). We further examined fiber cells of TW, and found that although the cell number per area was not significantly changed, the G-layer in the xylem fiber cell walls of transgenic plants was obviously thicker than that of WT (Figures 3A–J).

As the most typical feature of TW, G-layer is in rich of cellulose (Scurfield and Wardrop, 1963; Norberg and Meier, 1966; Cronshaw and Morey, 1968). Therefore, we determined the content of cellulose in the TW xylem of both WT and transgenic plants. Consistent with the thickened G-layer, the content of cellulose in the TW xylem of transgenic plants was significantly higher than that in the wild type (Figure 3K). We also confirmed the overexpression of *PtiCYP85A3* and accumulation of BZR1 protein in the TW xylem of transgenic plants. Again, consistent with the overexpression of *PtiCYP85A3*, BZR1 protein was more abundantly accumulated in the TW xylem of all transgenic plants than in that of WT plants (Figures 3L,M).

### TW Associated *PtiCesAs* Are Regulated by BR

**FIGURE 4:**
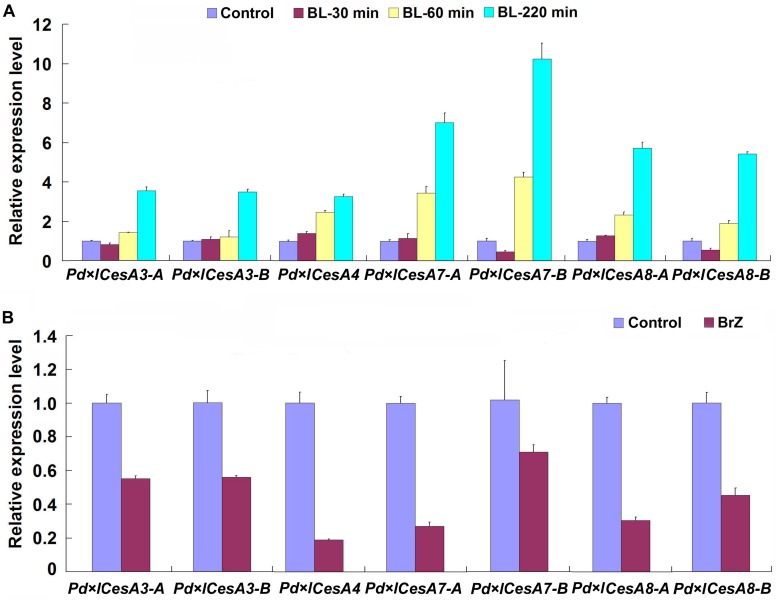
qRT-PCR analyses of cellulose biosynthesis genes. Stems of wild type Shangxin yang plants were treated with 100 nM 2, 4-epibrassinolide (EBL) for 0, 30, 60, 220 min, or with 5 mM brassinazole (Brz) for 10 days, and the relative expressions of *Pd* × *lCesAs* were determined. **(A)** Expressions of *Pd* × *lCesAs* in the stems treated with EBL. **(B)** Expressions of *Pd* × *lCesAs* in the tension wood xylem tissues of the stems treated with Brz. The relative expression of *Pd* × *lCesAs* was normalized using the housekeeping gene *Pd* × *lEF1*β. Gene expression value in the control was set to 1. Data were means ± SD (*n* = 6) of three independent biological replicates with three technical replicates each. Gene Potri ID numbers were provided in Supplementary Table S1.

In the genome of *Populus trichocarpa*, a total number of 17 cellulose biosynthesis genes were identified (Kumar et al., 2009). We examined the expression patterns of these *CesAs* during TW formation in Shanxin yang, and found that seven of them, *Pd* × *lCesA3-A*, *Pd* × *lCesA3-B*, *Pd* × *lCesA4*, *Pd* × *lCesA7-A*, *Pd* × *lCesA7-B*, *Pd* × *lCesA8-A*, and *Pd* × *lCesA8-B*, were highly expressed in the TW xylem tissues (Supplementary Figure S2). These results imply that these *CesA* genes may play an important role in the cellulose biosynthesis during G-layer formation. To confirm this hypothesis, the stems of WT plants were treated with 100 nM 2, 4-epibrassinolide (EBL) for different time periods, and the expression levels of these *CesA* genes were examined. As expected, expression of all these *CesA* genes were up-regulated by EBL (Figure 4A). We further tested the effect of brassinazole (Brz), a BR biosynthesis inhibitor, on the expression of these TW-induced *CesAs*. WT plants grown in greenhouse were inclined at 45° for 10 days and painted with 5 mM Brz on the upper side of stems. Then, the expression levels of these *CesAs* in TW xylem tissues were analyzed by qRT-PCR. Again, as expected, the transcription of these *CesA* genes were all down-regulated at different levels (Figure 4B). These results indicate that expressions of these TW associated *CesA* genes are regulated by BRs.

### Expression of TW Associated *CesAs* Are Up-Regulated in TW Xylem of *PtiCYP85A3* Transgenic Poplar

**FIGURE 5:**
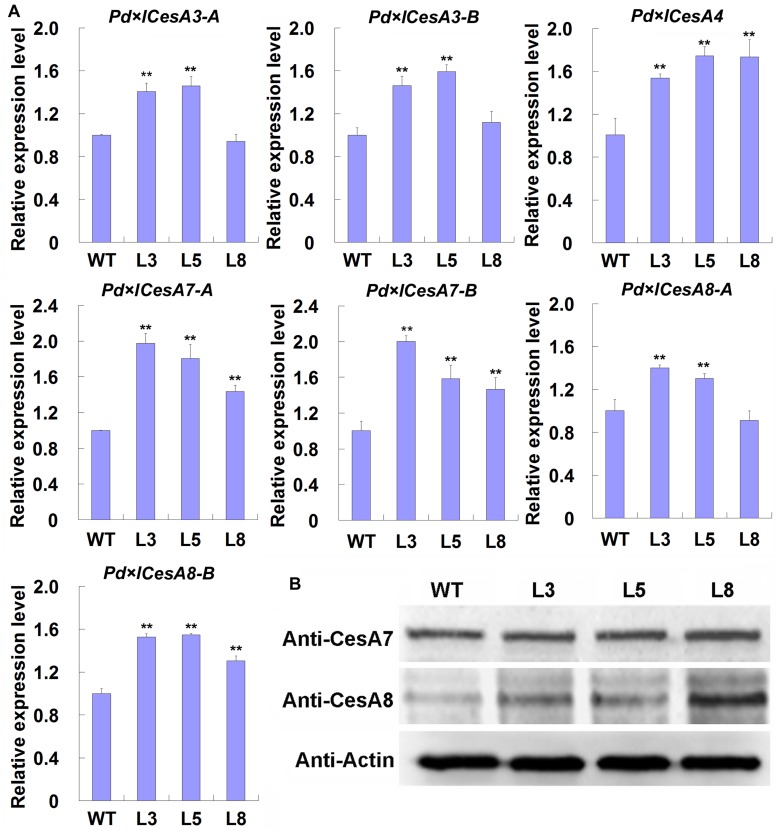
Expression analyses of *CesAs* in the TW xylem of wild type (WT) and transgenic Shanxin yang plants. **(A)** Transcription analyses of *CesAs.* Gene expression value in WT plants was set to 1. Data were means ± SD (*n* = 6) of three independent biological replicates with three technical replicates each. ** indicates a significant difference in comparison to WT at *P* < 0.01. **(B)** Western blotting analyses of proteins recognized by anti-CesA7 and anti-CesA8 antibodies in the TW xylem of WT and transgenic lines. M, protein molecular weight marker; WT, wide type; L3, L5, and L8, different transgenic lines overexpressing *PtiCYP85A3*. Anti-CesA7, AtCesA7 polyclonal antibody; Anti-CesA8, AtCesA8 polyclonal antibody; Anti-Actin, plant actin antibody. Gene Potri ID numbers were provided in Supplementary Table S1.

Based on the observations that TW associated *CesAs* were regulated by BRs, we speculated that the thickened G-layer in the TW fiber cell walls of transgenic plants maybe be due to the up-regulated expression of *CesA* genes. Therefore, we analyzed the expression levels of these *CesAs* in the TW xylem tissues of transgenic poplar plants overexpressing *PtiCYP85A3*. Indeed, all the tested *CesAs* were up-regulated in TW xylem tissues of transgenic plants (Figure 5A). We further checked the protein levels of CesA proteins recognized by AtCesA7 and AtCesA8 antibodies by Western blotting. Consistently, the levels of proteins recognized by both antibodies were significantly higher in the TW xylem of transgenic plants compared with that of WT plants (Figure 5B). These results imply that BRs could promote G-layer formation by regulating the expression of TW-associated *CesAs*.

### MYB128 Is Highly Expressed During TW Formation and Regulated by BR Signaling

**FIGURE 6:**
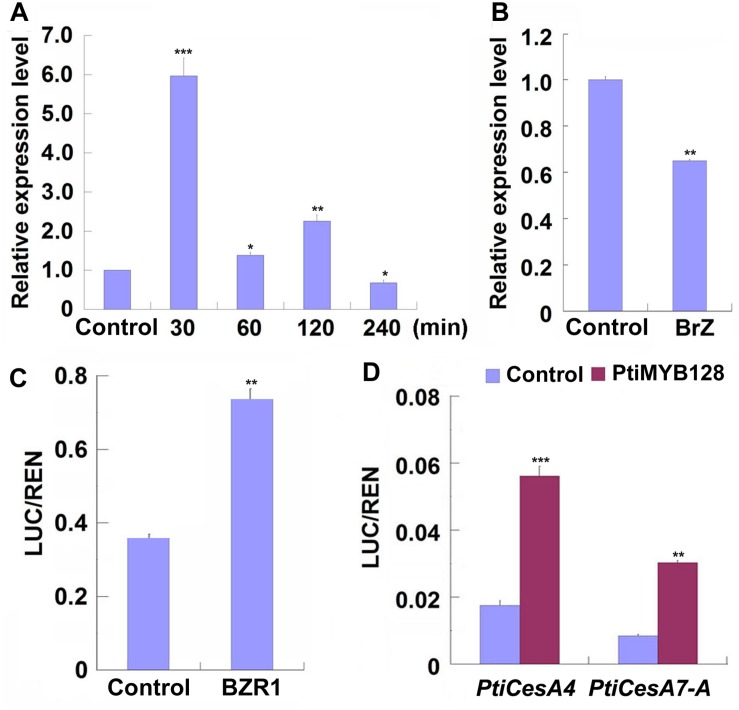
PtiMYB128 was regulated by BZR1 and positively regulates the expression of *PtiCesAs*. **(A,B)** *MYB128* was induced by EBL but suppressed by Brz. The transcription levels of *MYB128* in the TW xylem of wild type Shangxin yang (WT) stems treated with 100 nM EBL or 5 mM Brz were investigated. **(C)** BZR1 regulated the promoter of PtiMYB128. The control vector or pGreenII62-SK-*AtBZR1* were co-expressed with *35S*:*REN-ProPtiMYB128:LUC* in the leaf protoplasts of WT poplar. LUC/REN ratios represent the promoter activity of *PtiMYB128* activated by the Arabidopsis BZR1 (AtBZR1). Control, protoplasts co-transfected with pGreenII62-SK and *35S*:*REN-ProPtiMYB128:LUC*; BZR1, protoplasts co-transfected with pGreenII62-SK-AtBZR1 and *35S*:*REN-ProPtiMYB128:LUC*. **(D)** PtiMYB128 regulated the promoters of *PtiCesA4* and *PtiCesA7-A*. The control or pGreenII62-SK-*PtiMYB128* vector were co-expressed with the reporters *35S*:*REN-ProPtiCesA4:LUC* or *35S*:*REN-ProPtiCesA7-A:LUC* in the leaf protoplasts of WT poplar, respectively. LUC/REN ratios represent the promoter activity of *PtiCesA4* and *PtiCesA7-A* activated by PtiMYB128. Control, protoplasts co-transfected with pGreenII62-SK and each reporter; PtiMYB128, protoplasts co-transfected with pGreenII62-SK-*PtiMYB128* and each reporter. Data were means ± SD (*n* = 3) of three independent biological replicates with three technical replicates each. *, **, and *** indicate significant difference at *P* < 0.05, *P* < 0.01, and *P* < 0.001, respectively (Student’s *t*-test).

Previous studies have shown that poplar *CesAs*, including some TW-associated *CesAs*, participating in xylem development were regulated by a list of MYB transcription factors (Ohtani et al., 2011; Zhong et al., 2011; Lin et al., 2013; Ye and Zhong, 2015). We examined the expression patterns of xylem-related *MYBs* during TW formation in Shanxin yang plants and found that except *Pd* × *lMYB121*, all xylem-related *Pd* × *lMYBs* were highly expressed in the xylem tissues of TW or OW side. However, the expression levels of *Pd* × *lMYB10* and *Pd* × *lMYB128* were significantly higher in the TW xylem than in the OW xylem (Supplementary Figure S3). Further treatments with EBL or Brz demonstrated that both *Pd* × *lMYB10* and *Pd* × *lMYB128* were repressed by Brz, but only *Pd* × *lMYB128* was significantly induced by EBL (Figures 6A,B and Supplementary Figure S4). Therefore, TW-associated Pd × lMYB128 could be involved in BR-mediated G-layer formation in poplar.

We further investigated whether *Pd* × *lMYB128* was regulated by BR or BR signaling by transient transcription dual-luciferase assays. We found that AtBZR1, the key transcription factor in BR signaling, significantly activated the promoter of *PtiMYB128* (Figure 6C), suggesting that *Pd* × *lMYB128* could be regulated by BZR1 protein.

### *PtiCesAs* Are Regulated by PtiMYB128

Since the expression of *Pd* × *lMYB128* was responsive to EBL and Brz treatments, we further performed dual-luciferase assays to see if it can activate the promoters of TW-associated *PtiCesAs*. As we have expected, *PtiMYB128* successfully activated the promoters of *PtiCesA4* and *PtiCesA7-A*, showing a higher LUC/REN radio than the control (Figure 6D). Therefore, cellulose synthesis facilitated by *CesAs* in G-layer formation is regulated by PtMYB128.

## Discussion

As a class of plant specific polyhydroxylated steroid hormones, BRs have important functions in the growth and development, as well as in response to biotic and abiotic stresses of plants. In zinnias (*Zinnia elegans*), low concentration of BL promoted the differentiation of mesophyll cells into tracheary elements (Iwasaki and Shibaoka, 1991). In Cress plants (*Lepidium sativum*), Brz treatment inhibited the growth of secondary xylem (Nagata et al., 2001). In Arabidopsis, BR-deficient mutants displayed dwarfed phenotypes and abnormal development of vascular tissues, with excessively proliferated phloem cells and dramatically reduced xylem cells (Caño-Delgado et al., 2004, 2010; Xie et al., 2011; Hossain et al., 2012). However, the roles of BRs in TW formation are not well illustrated.

To date, among the more than 70 BRs identified in higher plants (Li et al., 1996), only BL and its immediate precursor castasterone (CS) have detectable biological activity (Yokota et al., 1982). The P450 protein CYP85A1 acts as a C-6 oxidase, which catalyzes the multiple C-6 oxidation reactions including 6-deoxo CS to CS (Bishop et al., 1999; Shimada et al., 2001). CYP85A1 and CYP85A3 in *Solanum lycopersicum*, AtCYP85A1 and AtCYP85A2 in *Arabidopsis thaliana*, CYP85A1 and CYP85A6 in *Pisum sativum*, BR6ox1 in *Vitis vinifera*, DWARF in *Oryza sativa* and *Hordeum vulgare*, and BRD1 (BRASSINOSTEROID DEFICIENT DWARF 1) in *Zea mays* and *Brachypodium distachyon*, were all involved in the multiple C-6 oxidation reactions, but only AtCYP85A2 in Arabidopsis and CYP85A3 in tomato could convert CS to BL (Bishop et al., 1996, 1999; Schultz et al., 2001; Shimada et al., 2001; Hong et al., 2002; Mori et al., 2002; Kim et al., 2005; Nomura et al., 2005; Symons et al., 2006; Jager et al., 2007; Gruszka et al., 2011; Makarevitch et al., 2012; Xu et al., 2015). In poplar, three BR-C6-oxidase encoding genes, named as *PtiCYP85A1*, *PtiCYP85A3*, and *PtiCYP85A4*, have been identified (Kim et al., 2008). *PtiCYP85A1* and *PtiCYP85A3* shared as high as 91.9 and 96.98% homology in DNA and amino acid sequence, respectively. The homologies of *PtiCYP85A4* and *PtiCYP85A3* were lower, 89.48% (DNA sequence) and 67.38% (protein sequence), respectively (Jin et al., 2017).

In order to understand the possible function of *PtiCYP85A3* in TW formation in poplar, we first examined the expression of genes involved in BR biosynthesis and metabolism, and the contents of CS and BL during TW formation in Shanxin yang (Figures 1A–H). *Pd* × *lCYP85A1* and *Pd* × *lCYP85A3* showed the same expression pattern, which was different from the expression pattern of *Pd* × *lCYP85A4* (Figures 1E,F). Therefore, *PtiCYP85A1* and *PtiCYP85A3* may have the same functions. The predominate expression of *Pd* × *lCYP85A3* in the TW-X tissue was accompanied with an increased CS content. Consistent with these observations, BZR1 protein was also abundantly accumulated in the xylem fiber cells undergoing G-layer development (Figures 2A,C). All these results imply that *PtiCYP85A3* is involved in TW formation, possibly by affecting the production and distribution of BR in poplar. This is further confirmed with transgenic poplar plants overexpressing *PtiCYP85A3*, which produced thicker G-layer with increased cellulose and BZR1 protein contents in the xylem fiber cell walls than did the WT plants (Figures 3A–M).

In Arabidopsis, BRs regulate cellulose biosynthesis by controlling the expression of CESA genes (Xie et al., 2011). Since the major part of TW fiber cell walls were composed of cellulose, we investigated the expressions of all the 17 cellulose biosynthesis genes identified in the poplar genome and found that seven of them were highly transcribed in the TW xylem (Supplementary Figure S2). Further studies indicated that their expressions were up-regulated by 2, 4-epibrassinolide (EBL) and down-regulated by brassinazole (Brz) (Figures 4A,B). Similar results were also observed in transgenic poplar plants overexpressing *PtiCYP85A3* (Figures 5A,B). These results demonstrate that BRs could improve G-layer formation by regulating *CesA* gene expression.

Previously, it was reported that *AtMYB103* was involved in secondary cell wall synthesis, and was predominantly expressed in TW than in opposite wood (Zhong et al., 2008; Chen et al., 2015). We found that expression of *Pd* × *lMYB128*, one of the homologs of *AtMYB103* in poplar, was up-regulated in the TW fiber cells and induced by EBL (Supplementary Figure S3 and Figure 6A). Based on the observations that *Pd* × *lMYB128* was induced by BR treatment and suppressed by BR biosynthesis inhibitor Brz, we deduced that it may be involved in BR signaling (Figures 6A,B). The poplar genome contains seven homologs of BZR1/BES1, a key positive transcriptional factor in Arabidopsis (Supplementary Figure S5). However, their possible functions are still unknown. Therefore, we performed dual-luciferase assays with the Arabidopsis BZR1, and found that BZR1 activated the promoter of *PtiMYB128*, which further activated the promoters of *PtiCesA4* and *PtCesA7-A* (Figures 6C,D). This is consistent with previous report that MYB128 could regulate the promoter activity of promoters of *PtrCesA4*, *PtrCesA8*, and *PtrCesA17*, the orthologs of *PtiCesA4*, *PtiCesA8-A*, and *PtiCesA7-B*, respectively (Zhong et al., 2011).

The released strain of growth stress was tightly correlation with the production of reaction wood (Yamamoto et al., 1991; Sugiyama et al., 1993; Okuyama et al., 1994). In TW, the longitudinally contractive released strain increased with cellulose content and crystallinity. It is negatively correlated with the Klason lignin content and microfibrillar angle (Sugiyama et al., 1993; Okuyama et al., 1994). The greater the contractive released strain was, the greater the tensile growth stress became, leading to the generation of thicker G-layers (Yoshida et al., 2002). Auxin, ethylene and gibberellin have been identified as important factors that regulate the formation of TW (Cronshaw and Morey, 1965; Morey and Cronshaw, 1968; Nakamura et al., 1994; Yoshida et al., 1999; Andersson-Gunnerås et al., 2003; Du and Yamamoto, 2003, 2007; Du et al., 2004; Clair et al., 2006; Funada et al., 2008; Love et al., 2009; Wang et al., 2017; Felten et al., 2018). Interestingly, most of the BR-related genes, except for the LRR receptor kinase precursors, were down-regulated in TW as compared to opposite wood after 6 h of mechanical bending treatment (Jin et al., 2011). Since the size of fiber cells in TW was much smaller than that in opposite wood (Jin and Kwon, 2009), the down-regulation of BR-related genes may be correlated with the reduced size of fiber cells in TW, thereby implying the possible involvement of BR in TW development (Jin et al., 2011). However, the molecular mechanism how BRs regulate TW formation still lacks accuracy. Our study demonstrated, for the first time, that BRs increased the thickness of G-layer by regulating the expression of *PtiMYB128*, one of the homologous genes of *AtMYB103*, to activate the expression of cellulose synthase genes, and consequently affected the formation of TW in poplar. A model was proposed to illustrate the biological roles of BRs in TW formation in *Populus* (Supplementary Figure S6). Under the action of external force or gravity, some secondary signal activated the biosynthetic and signal transduction pathways of BRs, thus more activated BZR1/BES1 moved into the nucleus to promote the transcription of *PtiMYB128*, which up-regulated the expressions of TW-associated cellulose synthesis genes, and as a result, facilitated the thickness of G-layers during TW formation. Taken together, our findings provide a new insight into the biological functions of *PtiCYP85A3* in the TW formation in poplar.

## Conclusion

In this study, we demonstrated that *PtiCYP85A3* plays a pivotal role in TW formation in poplar. Overexpression of *PtiCYP85A3* promoted G-layer formation, accompanied with augmented BZR1 accumulation and up-regulated *CesA* gene expression, in the xylem of the TW in transgenic plants. In addition, *CesA* gene promoters were promoted by PtiMYB128, which was activated by BZR1.

## Data Availability Statement

All datasets generated for this study are included in the article/Supplementary Material.

## Author COntributions

YJ, CY, CJ, XG, BL, CW, and FK performed the experiments and analyzed the data. YJ, HZ, and HW conceived the study. YJ, CY, HZ, and HW wrote the manuscript. All authors read and agreed at the last version of the manuscript.

## Conflict of Interest

The authors declare that the research was conducted in the absence of any commercial or financial relationships that could be construed as a potential conflict of interest.

## Footnotes

1 http://www.genomics.org.cn

2 http://www.agrisera.com

3 http://www.immunogen.com.cn

## References

[B1] Altmann T. (1999). Molecular physiology of brassinosteroids revealed by the analysis of mutants. *Planta* 208 1–11. 10.1007/s004250050528 10212999

[B2] Andersson-Gunnerås S. Hellgren J. M. Bjorklund S. Regan S. Moritz M. Sundberg B. (2003). Asymmetric expression of a poplar ACC oxidase controls ethylene production during gravitational induction of tension wood. *Plant J.* 34 339–349. 10.1046/j.1365-313x.2003.01727.x 12713540

[B3] Baba K. Adachi K. Take T. Yokoyama T. Itoh T. Nakamura T. (1995). Induction of tension wood in GA3-treated branches of the weeping type of Japanese cherry, *Prunus spachiana*. *Plant Cell Physiol.* 36 983–988. 10.1093/oxfordjournals.pcp.a078870

[B4] Bishop G. J. Harrison K. Jones J. D. (1996). The tomato Dwarf gene isolated by heterologous transposon tagging encodes the first member of a new cytochrome P450 family. *Plant Cell* 8 959–969. 10.1105/tpc.8.6.959 8672892 PMC161151

[B5] Bishop G. J. Nomura T. Yokota T. Harrison T. Noguchi T. Fujioka S. (1999). The tomato DWARF enzyme catalyses C-6 oxidation in brassinosteroid biosynthesis. *Proc. Natl. Acad. Sci. U.S.A.* 96 1761–1766. 10.1073/pnas.96.4.1761 9990098 PMC15587

[B6] Caño-Delgado A. Lee J. Y. Demura T. (2010). Regulatory mechanisms for specification and patterning of plant vascular tissues. *Annu. Rev. Cell. Dev. Biol.* 26 605–637. 10.1146/annurev-cellbio-100109-104107 20590454

[B7] Caño-Delgado A. Yin Y. Yu C. Vafeados D. Mora-Garcia S. Cheng J.-C. (2004). BRL1 and BRL3 are novel brassinosteroid receptors that function in vascular differentiation in *Arabidopsis*. *Development* 131 5341–5351. 10.1242/dev.01403 15486337

[B8] Chen J. Chen B. Zhang D. (2015). Transcript profiling of *Populus tomentosa* genes in normal, tension, and opposite wood by RNA-seq. *BMC Genomics* 16:164. 10.1186/s12864-015-1390-y 25886950 PMC4372042

[B9] Choe S. Dilkes B. P. Fujioka S. Takatsuto S. Sakurai A. Feldmann K. A. (1998). The *DWF4* gene of *Arabidopsis* encodes a cytochrome P450 that mediates multiple 22 alpha-hydroxylation steps in brassinosteroid biosynthesis. *Plant Cell* 10 231–243. 10.1105/tpc.10.2.231 9490746 PMC143988

[B10] Clair B. Alméras T. Pilate G. Jullien D. Sugiyama J. Riekel C. (2010). Maturation stress generation in poplar tension wood studied by synchrotron radiation microdiffraction. *Plant Physiol.* 155 562–570. 10.1104/pp.110.167270 21068364 PMC3075793

[B11] Clair B. Ruelle J. Beauchene J. Prevost M. F. Fournier M. (2006). Tension wood and opposite wood in 21 tropical rain forest species 1. Occurrence and effciency of the G-layer. *IAWA J.* 27 329–338. 10.1163/22941932-90000158

[B12] Coutand C. Fournier M. Moulia B. (2007). The gravitropic response of poplar trunks: key role of prestressed wood regulation and the relative kinetics of cambial growth versus wood maturation. *Plant Physiol.* 144 1166–1180. 10.1104/pp.106.088153 17468227 PMC1914190

[B13] Cronshaw J. Morey P. R. (1965). Induction of tension wood by 2,3,5-tri-iodobenzoic acid. *Nature* 205 816–818. 10.1038/205816a0

[B14] Cronshaw J. Morey P. R. (1968). The effect of plant growth substances on the development of tension wood in horizontally inclined stem of *Acer rubrum seedlings*. *Protoplasma* 65 379–391. 10.1007/bf01666298

[B15] Déjardin A. Laurans F. Arnaud D. Breton C. (2010). Wood formation in angiosperms. *C. R. Biol.* 333 325–334. 10.1016/j.crvi.2010.01.010 20371107

[B16] Desprez T. Juraniec M. Crowell E. F. Jouy H. Pochylova Z. Parcy F. (2007). Organization of cellulose synthase complexes involved in primary cell wall synthesis in *Arabidopsis thaliana*. *Proc. Natl. Acad. Sci. U.S.A.* 104 15572–15577. 10.1073/pnas.0706569104 17878303 PMC2000492

[B17] Du S. Uno H. Yamamoto F. (2004). Roles of auxin and gibberellin in gravityinduced tension wood formation in *Aesculus turbinata* seedlings. *IAWA J.* 25 337–347. 10.1163/22941932-90000370

[B18] Du S. Yamamoto F. (2003). Ethylene evolution changes in the stem of *Metasequoia glyptostroboides* and *Aesculus turbinata* seedlings in relation to gravity-induced reaction wood formation. *Trees* 17 522–528. 10.1007/s00468-003-0275-x

[B19] Du S. Yamamoto F. (2007). An overview of the biology of reaction wood formation. *J. Integr. Plant Biol.* 49 131–143. 10.1111/j.1744-7909.2007.00427.x

[B20] Felten J. Vahala J. Love J. Gorzsás A. Rüggeberg M. Delhomme N. (2018). Ethylene signaling induces gelatinous layers with typical features of tension wood in hybrid aspen. *New Phytol.* 218 999–1014. 10.1111/nph.15078 29528503

[B21] Foster C. E. Martin T. M. Pauly M. (2010). Comprehensive compositional analysis of plant cell walls (lignocellulosic biomass). Part I: lignin. *J. Vis. Exp.* 37:e1745. 10.3791/1745 PMC3144576 20224547

[B22] Funada R. Miura T. Shimizu Y. Kinase T. Nakaba S. Kubo T. (2008). Gibberellin-induced formation of tension wood in angiospermae trees. *Planta* 227 1409–1414. 10.1007/s00425-008-0712-6 18320214

[B23] Gorshkova T. Mokshina N. Chernova T. Ibragimova N. Salnikov V. Mikshina P. (2015). Aspen tension wood fibers contain beta-(1→4)-galactans and acidic arabinogalactans retained by cellulose microfibrils in gelatinous walls. *Plant Phytol.* 169 2048–2063. 10.1104/pp.15.00690 PMC4634055 26378099

[B24] Goswami L. Dunlop J. W. C. Jungnikl K. Eder M. Gierlinger N. Coutand C. (2008). Stress generation in the tension wood of poplar is based on the lateral swelling power of the G-layer. *Plant J.* 56 531–538. 10.1111/j.1365-313x.2008.03617.x 18643995

[B25] Gruszka D. Szarejko I. Maluszynski M. (2011). Identification of barley *DWARF* gene involved in brassinosteroid synthesis. *Plant Growth Regul.* 65 343–358. 10.1007/s10725-011-9607-9

[B26] Guedes F. T. P. Laurans F. Quemener B. Assor C. Lainé-Prade V. Boizot N. (2017). Non-cellulosic polysaccharide distribution during G-layer formation in poplar tension wood fibers: abundance of rhamnogalacturonan I and arabinogalactan proteins but no evidence of xyloglucan. *Planta* 246 857–878. 10.1007/s00425-017-2737-1 28699115

[B27] Hellens R. P. Allan A. C. Friel E. N. Bolitho K. Grafton K. Templeton M. D. (2005). Transient expression vectors for functional genomics, quantification of promoter activity and RNA silencing in plants. *Plant Methods* 1 13–26. 16359558 10.1186/1746-4811-1-13 PMC1334188

[B28] Hellgren J. M. Olofsson K. Sundberg B. (2004). Patterns of auxin distribution during gravitational induction of reaction wood in poplar and pine. *Plant Physiol.* 135 212–220. 10.1104/pp.104.038927 15122024 PMC429355

[B29] Hong Z. Ueguchi-Tanaka M. Shimizu-Sato S. Inukai Y. Fujioka S. Shimada Y. (2002). Loss-of-function of a rice brassinosteroid biosynthetic enzyme, C-6 oxidase, prevents the organized arrangement and polar elongation of cells in the leaves and stem. *Plant J.* 32 495–508. 10.1046/j.1365-313x.2002.01438.x 12445121

[B30] Hossain Z. McGarvey B. Amyot L. Gruber M. Jung J. Hannoufa A. (2012). DIMINUTO 1 affects the lignin profile and secondary cell wall formation in *Arabidopsis*. *Planta* 235 485–498. 10.1007/s00425-011-1519-4 21947665

[B31] Iwasaki T. Shibaoka H. (1991). Brassinosteroids act as regulators of tracheary-element differentiation in isolated *Zinnia* mesophyll cells. *Plant Cell Physiol.* 32 1007–1014. 10.1093/oxfordjournals.pcp.a078163

[B32] Jager C. E. Symons G. M. Nomura T. Yamada Y. Smith J. J. Yamaguchi S. (2007). Characterization of two brassinosteroid C-6 oxidase genes in pea. *Plant Physiol.* 143 1894–1904. 10.1104/pp.106.093088 17322341 PMC1851809

[B33] Jin H. Do J. Moon D. Noh E. W. Kim W. Kwon M. (2011). EST analysis of functional genes associated with cell wall biosynthesis and modification in the secondary xylem of the yellow poplar (*Liriodendron tulipifera*) stem during early stage of tension wood formation. *Planta* 234 959–977. 10.1007/s00425-011-1449-1 21688015

[B34] Jin H. Kwon M. (2009). Mechanical bending-induced tension wood formation with reduced lignin biosynthesis in *Liriodendron tulipifera*. *J. Wood Sci.* 55 401–408. 10.1007/s10086-009-1053-1

[B35] Jin Y. L. Tang R. J. Wang H. H. Jiang C. M. Bao Y. Yang Y. (2017). Overexpression of *Populus trichocarpa CYP85A3* promotes growth and biomass production in transgenic trees. *Plant Biotechnol. J.* 15 1309–1321. 10.1111/pbi.12717 28258966 PMC5595715

[B36] Jourez B. Avella-Shaw T. (2003). Effect of gravitational stimulus duration on tension wood formation in young stems of poplar (*P-euramericana ev ‘Ghoy’*). *Ann. For. Sci.* 60 31–41. 10.1051/forest:2002071

[B37] Jourez B. Riboux A. Leclercq A. (2001). Anatomical characteristics of tension wood and opposite wood in young inclined stem of poplar (*Populus euramericana cv ‘Ghoy’*). *IAWA J.* 22 133–157. 10.1163/22941932-90000274

[B38] Kim B. K. Fujioka S. Takatsuto S. Tsujimoto S. Choe S. (2008). Castasterone is a likely end product of brassinosteroid biosynthetic pathway in rice. *Biochem. Biophys. Res. Commun.* 374 614–619. 10.1016/j.bbrc.2008.07.073 18656444

[B39] Kim T. W. Hwang J. Y. Kim Y. S. Joo S. H. Chang S. C. Lee J. S. (2005). *Arabidopsis CYP85A2*, a cytochrome P450, mediates the baeyer-villiger oxidation of castasterone to brassinolide in brassinosteroid biosynthesis. *Plant Cell* 17 2397–2412. 10.1105/tpc.105.033738 16024588 PMC1182497

[B40] Kumar M. Thammannagowda S. Bulone V. Chiang V. Han K. H. Joshi C. P. (2009). An update on the nomenclature for the cellulose synthase genes in *Populus*. *Trends Plant Sci.* 14 248–254. 10.1016/j.tplants.2009.02.004 19375973

[B41] Kwon M. (2008). Tension wood as model system to explore the carbon partitioning between lignin and cellulose biosynthesis in woody plants. *J. Appl. Biol. Chem.* 51 83–87. 10.3839/jabc.2008.018

[B42] Li J. M. Nagpal P. Vitart V. McMorris T. C. Chory J. (1996). A role for brassinosteroids in light-dependent development of *Arabidopsis*. *Science* 272 398–401. 10.1126/science.272.5260.398 8602526

[B43] Lin Y. C. Li W. Sun Y. H. Kumari S. Wei H. Li Q. (2013). SND1 transcription factor-directed quantitative functional hierarchical genetic regulatory network in wood formation in *Populus trichocarpa*. *Plant Cell* 25 4324–4341. 10.1105/tpc.113.117697 24280390 PMC3875721

[B44] Little C. H. A. Pharis R. P. (1995). “Hormonal control of radial and longitudinal growth in the tree stem,” in *Plant Stems*, ed. Gartner B. L. (San Diego: Academic Press), 281–319. 10.1016/b978-012276460-8/50015-1

[B45] Love J. Björklund S. Vahala J. Hertzberg M. Kangasjorvi J. Sundberg B. (2009). Ethylene is an endogenous stimulator of cell division in the cambial meristem of *Populus*. *Proc. Natl. Acad. Sci. U.S.A.* 106 5984–5989. 10.1073/pnas.0811660106 19293381 PMC2657089

[B46] Makarevitch I. Thompson A. Muehlbauer G. J. Springer N. M. (2012). Brd1 gene in maize encodes a brassinosteroid C-6 oxidase. *PLoS One* 7:e30798. 10.1371/journal.pone.0030798 22292043 PMC3266906

[B47] Mellerowicz E. J. Baucher M. Sundberg B. Boerjan W. (2001). Unravelling cell wall formation in the woody dicot stem. *Plant Mol. Biol.* 47 239–274. 10.1007/978-94-010-0668-2_15 11554475

[B48] Morey P. R. Cronshaw J. (1968). Developmental changes in the secondary xylem of *Acer rubrum* induced by gibberellic acid, various auxins and 2,3,5-tri-iodobenzoic acid. *Protoplasma* 65 315–326. 10.1007/bf01682535

[B49] Mori M. Nomura T. Ooka H. Ishizaka M. Yokota T. Sugimoto K. (2002). Isolation and characterization of a rice *dwarf* mutant with a defect in brassinosteroid biosynthesis. *Plant Physiol.* 130 1152–1161. 10.1104/pp.007179 12427982 PMC166636

[B50] Moyle R. Schrader J. Stenberg A. Olsson O. Saxena S. Sandberg G. (2002). Environmental and auxin regulation of wood formation involves members of the Aux/IAA gene family in hybrid aspen. *Plant J.* 31 675–685. 10.1046/j.1365-313x.2002.01386.x 12220260

[B51] Murashige T. Skoog F. (1962). A revised medium for rapid growth and bioassays with tobacco tissue cultures. *Plant Physiol.* 15 473–495.

[B52] Nagata N. Asami T. Yoshida S. (2001). Brassinazole, an inhibitor of brassinosteroid biosynthesis, inhibits development of secondary xylem in cress plants (*Lepidium sativum*). *Plant Cell Physiol.* 42 1006–1011. 10.1093/pcp/pce122 11577196

[B53] Nakamura T. Saotome T. Ishiguro Y. Itoh R. Higurashi S. Hosono M. (1994). The effects of GA3 on weeping of growing shoots of the Japanese cherry, *Prunus spachiana*. *Plant Cell Physiol.* 35 523–527.

[B54] Nomura T. Kushiro T. Yokota T. Kamiya Y. Bishop G. J. Yamaguchi S. (2005). The last reaction producing brassinolide is catalyzed by cytochrome P-450s, *CYP85A3* in tomato and *CYP85A2* in *Arabidopsis*. *J. Biol. Chem.* 280 17873–17879. 10.1074/jbc.m414592200 15710611

[B55] Norberg P. H. Meier H. (1966). Physical and chemical properties of the gelatinous layer in tension wood fibres of Aspen (*Populus tremula L*). *Holzforschung* 20 174–178. 10.1515/hfsg.1966.20.6.174

[B56] Ohtani M. Nishikubo M. Xu B. Yamaguchi M. Mitsuda N. Goue N. (2011). A NAC domain protein family contributing to the regulation of wood formation in poplar. *Plant J.* 67 499–512. 10.1111/j.1365-313x.2011.04614.x 21649762

[B57] Okuyama T. Yamamoto H. Yoshida M. Hattori Y. Archer R. R. (1994). Growth stresses in tension wood: role of microfibrils and lignification. *Ann. For. Sci.* 51 291–300. 10.1051/forest:19940308

[B58] Persson S. Paredez A. Carroll A. Palsdottir H. Doblin M. Poindexter P. (2007). Genetic evidence for three unique components in primary cell-wall cellulose synthase complexes in *Arabidopsis*. *Proc. Natl. Acad. Sci. U.S.A.* 104 15566–15571. 10.1073/pnas.0706592104 17878302 PMC2000526

[B59] Pilate G. Dejardin A. Laurans F. Leple J. C. (2004). Tension wood as a model for functional genomics of wood formation. *New Phytol.* 164 63–72. 10.1111/j.1469-8137.2004.01176.x 33873474

[B60] Ruelle J. Clair B. Beauchene J. Prevost M. F. Fournier M. (2006). Tension wood and opposite wood in 21 tropical rain forest species 2. Comparison of some anatomical and ultrastructural criteria. *IAWA J.* 27 341–376. 10.1163/22941932-90000159

[B61] Saleh A. Lumbreras V. Lopez C. Dominguez-Puigjaner E. Kizis D. Pagès M. (2006). Maize DBF1 interactor protein 1 containing an R3H domain is a potential regulator of DBF1 activity in stress responses. *Plant J.* 46 747–757. 10.1111/j.1365-313x.2006.02742.x 16709191

[B62] Schultz L. Kerckhoffs L. H. Klahre U. Yokota T. Reid J. B. (2001). Molecular characterization of the brassinosteroid-deficient lkb mutant in pea. *Plant Mol. Biol.* 47 491–498. 11669574 10.1023/a:1011894812794

[B63] Scurfield G. Wardrop A. B. (1963). The nature of reaction wood VII. Lignification in reaction wood. *Aust. J. Bot.* 11 107–116.

[B64] Shimada Y. Fujioka S. Miyauchi N. Kushiro M. Takatsuto S. Nomura T. (2001). Brassinosteroid-6-oxidases from *Arabidopsis* and tomato catalyze multiple C-6 oxidation in brassinosteroid biosynthesis. *Plant Physiol.* 126 770–779. 10.1104/pp.126.2.770 11402205 PMC111167

[B65] Srebotnik E. Messner K. (1994). A simple method that uses differential staining and light microscopy to assess the selectivity of wood delignification by white rot fungi. *Appl. Environ. Microbiol.* 60 1383–1386. 10.1128/aem.60.4.1383-1386.1994 16349245 PMC201488

[B66] Sugiyama K. Okuyama T. Yamamoto H. Yoshida M. (1993). Generation process of growth stress in cell walls: relation between longitudinal released strain and chemical composition. *Wood Sci. Technol.* 27 257–262.

[B67] Symons G. M. Davies C. Shavrukov Y. Dry I. B. Reid J. B. Thomas M. R. (2006). Grapes on steroids. Brassinosteroids are involved in grape berry ripening. *Plant Physiol.* 140 150–158. 10.1104/pp.105.070706 16361521 PMC1326039

[B68] Szekeres M. Németh K. Koncz-Kálmán Z. Mathur J. Kauschmann A. Altmann T. (1996). Brassinosteroids rescue the deficiency of CYP90, a cytochrome P450, controlling cell elongation and de-etiolation in *Arabidopsis*. *Cell* 85 171–182. 10.1016/s0092-8674(00)81094-6 8612270

[B69] Taylor N. G. Howells R. M. Huttly A. K. Vickers K. Turner S. R. (2003). Interactions among three distinct CesA proteins essential for cellulose synthesis. *Proc. Natl. Acad. Sci. U.S.A.* 100 1450–1455. 10.1073/pnas.0337628100 12538856 PMC298793

[B70] Timell T. E. (1969). The chemical composition of tension wood. *Nord. Pulp Pap. Res. J.* 72 173–181.

[B71] Turk E. M. Fujioka S. Seto H. Shimada Y. Takatsuto S. Yoshida S. (2005). BAS1 and SOB7 act redundantly to modulate *Arabidopsis* photomorphogenesis via unique brassinosteroid inactivation mechanisms. *Plant J.* 42 23–34. 10.1111/j.1365-313x.2005.02358.x 15773851

[B72] Wang H. H. Jiang C. M. Wang C. T. Yang Y. Yang L. Gao X. Y. (2015). Antisense expression of the fasciclin-like arabinogalactan protein PtFLA6 gene in Populus inhibits expression of its homologous genes and alters stem biomechanics and cell wall composition in transgenic trees. *J. Exp. Bot.* 66 1291–1302. 10.1093/jxb/eru479 25428999 PMC4339592

[B73] Wang H. H. Jin Y. L. Wang C. T. Li B. Jiang C. M. Sun Z. (2017). Fasciclin-like arabinogalactan proteins, PtFLA6, play important roles in GA-mediated tension wood formation in *Populus*. *Sci. Rep.* 7:6182. 10.1038/s41598-017-06473-9 PMC5522414 28733593

[B74] Wang H. H. Tang R. J. Liu H. Chen H. Y. Liu J. Y. Jiang X. N. (2013). Chimeric repressor of *PtSND2* severely affects wood formation in transgenic populus. *Tree Physiol.* 33 878–886. 10.1093/treephys/tpt058 23939552

[B75] Xie L. Yang C. Wang X. (2011). Brassinosteroids can regulate cellulose biosynthesis by controlling the expression of *CESA* genes in *Arabidopsis*. *J. Exp. Bot.* 62 4495–4506. 10.1093/jxb/err164 21617247 PMC3170551

[B76] Xu Y. Zhang X. Li Q. Cheng Z. Lou H. Ge L. (2015). BdBRD1, a brassinosteroid C-6 oxidase homolog in brachypodium distachyon L., is required for multiple organ development. *Plant Physiol. Biochem.* 86 91–99. 10.1016/j.plaphy.2014.11.018 25438141

[B77] Yamamoto H. Okuyama T. Yoshida M. Sugiyama K. (1991). Generation process of growth stresses in cell walls. III. Growth stress in compression wood. *Mokuzai Gakkai Shi* 37 94–100.

[B78] Yamamoto H. Yoshida M. Okuyama T. (2002). Growth stress controls negative gravitropism in woody plant stems. *Planta* 216 280–292. 10.1007/s00425-002-0846-x 12447542

[B79] Ye Z. H. Zhong R. (2015). Molecular control of wood formation in trees. *J. Exp. Bot.* 66 4119–4131. 25750422 10.1093/jxb/erv081

[B80] Yokota T. Arima M. Takahashi N. (1982). Castasterone, a new phytosterol with plant-hormone potency, from chestnut insect gall. *Tetrahedron Lett.* 23 1275–1278. 10.1016/s0040-4039(00)87081-1

[B81] Yoshida M. Nakamura T. Yamamoto H. Okuyama T. (1999). Negative gravitropism and growth stress in GA3-treated branches of *Prunus spachiana* Kitamura f. *spachiana cv. Plenarosea*. *J. Wood Sci.* 45 368–372. 10.1007/bf01177907

[B82] Yoshida M. Ohta H. Okuyama T. (2002). Tensile growth stress and lignin distribution in the cell walls of black locust (*Robinia pseudoacacia*). *J Wood Sci.* 48 99–105. 10.1007/bf00767285

[B83] Yoshida M. Okuda T. Okuyama T. (2000). Tension wood and growth stress induced by artificial inclination in *Liriodendron tulipifera* Linn. and *Prunus spachiana* Kitamura f. ascendens Kitamura. *Ann. For. Sci.* 57 739–746. 10.1051/forest:2000156

[B84] Zhang X. Dominguez P. G. Kumar M. Bygdell J. Miroshnichenko S. Sundberg B. (2018). Cellulose synthase stoichiometry in aspen differs from *Arabidopsis* and Norway Spruce. *Plant Physiol.* 177 1096–1107. 10.1104/pp.18.00394 29760198 PMC6053019

[B85] Zhong R. Lee C. Zhou J. McCarthy R. L. Ye Z. H. (2008). A battery of transcription factors involved in the regulation of secondary cell wall biosynthesis in *Arabidopsis*. *Plant Cell* 10 2763–2782. 10.1105/tpc.108.061325 18952777 PMC2590737

[B86] Zhong R. McCarthy R. L. Lee C. Ye Z. H. (2011). Dissection of the transcriptional program regulating secondary wall biosynthesis during wood formation in poplar. *Plant Physiol.* 157 1452–1468. 10.1104/pp.111.181354 21908685 PMC3252164

