# Supplementary material for: PtiCYP85A3, a BR C-6 Oxidase Gene, Plays a Critical Role in Brassinosteroid-Mediated Tension Wood Formation in Poplar
Source: Front Plant Sci. 2020 Apr 24;11:468. doi: 10.3389/fpls.2020.00468 (PMC7193022; doi:10.3389/fpls.2020.00468)
Supplement: Supplementary file 1 [file Data_Sheet_1.PDF]

Title: *PtiCYP85A3*, a BR C-6 oxidase gene, plays a critical role in brassinosteroid-mediated tension wood formation in poplar

Authors: Yanli Jin[1,2,4,†], Chunyan Yu[1,4,†], Chunmei Jiang[1,5], Xiaotong Guo[1,4], Bei Li[1,4], Cuiting Wang[2], Fanjing Kong[3], Hongxia Zhang[1,2,4,*] and Haihai Wang[2,*]

Supplementary materials

Table S1. Primer sequences used in this study.

Figure S1. The area ratio of TW to OW in wild type (WT) and transgenic Shanxin yang plants overexpressing *PtiCYP85A3*.

Figure S2. The expression patterns of *CesAs* during TW formation in wild type Shanxin yang plants.

Figure S3. The expression patterns of xylem-related *MYBs* during TW formation in wild type Shanxin yang plants.

Figure S4. The relative expression of *MYB10* in wild type Shanxin yang plants treated with eBL and BrZ.

Figure S5. The expression patterns of putative poplar *BESs* during TW formation in wild type Shanxin yang plants.

Figure S6. A proposed model of BR regulated G-layer formation in poplar.

Table S1. Primer sequences used in this study.

Gene	Identifier	Forward Primer (5' to 3')	Reverse Primer (5' to 3')
**RT-PCR primers**			
PtiCYP85A3	Potri.017G099000.1	AATTAGAGAAAAGAAAAGGCCTGAG	CCTTTCCTGGACACTGCCTGGTACCT
PtiCYP85A1	Potri.004G117700.1	AGCGCAAGGTATGGGAGTTTTTTCAA	TGGGGCTGATGAGGGATAACAATG
PtiEF1β	Potri.009G018600.1	GACAAGAAGGCAGCGGAGGAGAG	CAATGAGGGAATCCACTGACACAAG
PtiCesA6-D	Potri.002G066600.1	ATGATGGTGCGGCCATGCTTACTT	TTCTGGAGCTCGAGGTTCAATGCT
PtiCesA3-D	Potri.001G266400.1	GTCTGAGACTTCAGAGTTTGCCAGGA	TGTGCAAAGTACCATTCTGGAGCC
PtiCesA4	Potri.002G257900.1	CTGGATCCTTGACCAGTTCCCAAA	ATTAGGCTCACCCTCACGCTCAAA
PtiCesA6-C	Potri.005G194200.1	CTGCAAACACAGTCTTGTCCATCC	AGCTTCGAAAGTAAGCATGGCTGC
PtiCesA6-A	Potri.005G087500.1	TCTCGCGGTTGATTATCCTGTGGA	AGACGTCTCGGAGAGAGCTTCAAA
PtiCesA7-A	Potri.006G181900.1	CTTCCATGTGCACCTTTGAAGCCA	TCAGGAGCTCGAGGTTCTATGCTA
PtiCesA8-A	Potri.011G069600.1	TGACCCACTGAAAGAGCCTCCATT	GTAAGCATGGCAGCACCATCATCA
PtiCesA6-B	Potri.007G076500.1	ACTGATCACTGCGAACACTGTCCT	TTCAAATGTGAGCATGGCAGCACC
PtiCesA1-A	Potri.018G029400.1	ACTTGCTGTTGATTACCCTGTCG	AAAGGCACCCACTTCCTTGCAAAC
PtiCesA1-B	Potri.006G251900.1	CCATTCTTGCTGTTGATTACCCTGTCG	CCACTTCCTTGCAAATTCCGCAGT
PtiCesA3-A	Potri.006G052600.1	GAGAAAGAAGGAGAGCCGTCTCAA	ACAGTATTGGCTGTGACCAGTGGA

PtiCesA3-C	Potri.009G060800.1	GATGATGGTGCTGCAATGTTGACG	ATTCTGGAGCCCGAGGTTCAATGT
PtiCesA3-B	Potri.016G054900.1	AGAAAGAAGGAGAGCCGTCTCAGT	ACTGTATTGGCTGTGACCAGTGGA
PtiCesA6-E	Potri.013G019800.1	CAACAGACACGATATGCAGCACCA	AAAGGATGATGAAGGTCGTGAGGC
PtiCesA6-F	Potri.005G027600.1	TGCTGCTATGCTGACATTTGAGGC	GGTGCCCGAGGTTCAATGCTAAAT
PtiCesA7-B	Potri.018G103900.1	CTCTAGTCACGGGCAACACACTTT	GTGCACATTGAAGCACCATCGTCA
PtiCesA8-B	Potri.004G059600.1	ACCGCCATTGATCACTGCCAATAC	AGCTGCACCATCATCAGACACGTA
PtiBES1-1	Potri.014G041600.1	CGCCTGTAACCCCACCTCTCTC	TCGCGCCAAAGAAACAAACTG
PtiBES1-2	Potri.002G133700.1	GCCGTCGTGGAGGGAGAGAGAGA	CGGGCTGGGAAACGATGAAGATA
PtiBES1-3	Potri.007G030700.1	GGGGAGAGGTGCGGAGTTTGA	CGCAAGACCATCCATCCCAATTA
PtiBES1-5	Potri.004G062400.1	CGACGTGGAAGGAGCGAGAGAAC	AGGGCTTGGATTATAGGATGCACATG
PtiBES1-6	Potri.011G071800.1	CGACGTGGAAGGAGCGAGAGAAC	CCACCTATAATATCCATGCGTTCCACAG
PtiBES1-7	Potri.003G026600.1	CTGGGCTAAGAATGTATGGCAACTA	GCTGGACTGGGAAAGGAAGAGGATC
PtiBES1-8	Potri.001G386900.1	CGGCATCTTCATCAGCTTCCTCCT	GAGGCAACTAGGCTAAATGTGGGAGAC
PtiCPD	Potri.010G189800.1	TTCTTTGTGGTTCTCCCGGCGATAGT	CGGTTCAGGGTTCTCGGTCTTGTATG
PtiDWF4	Potri.007G026500.1	GCGAAAGATGGAGGAGAGAATTGCTG	CGTGGCCAGCAAAGAGCAAGC
PtiBAS1	Potri.006G154500.1	TGGCAGGTGCAACGTTTCTAGT	GCTAGTTCCGGTGGTGGCAA

PtiMYB2	Potri.001G258700.1	TTGGAGTGATGTAGCAAGGAA	GATGAAGATGACAGTGACGGAT
PtiMYB3	Potri.001G267300.1	CCGCTATGATGTGAGTGGTGC	GTTGTCGTTGCTGCTGCTTTT
PtiMYB10	Potri.001G099800.1	CGGCTATGGATGTTGGAGTGAGGTTC	ATCGGTTCCGGTAGTTGCATTGGTTAT
PtiMYB18	Potri.004G086300.1	CAATGTTGCTGGAGAGCTGTT	CAGGTTGATGAAGGAGAAGGTC
PtiMYB74	Potri.015G082700.1	AGCTGGTCTGCAAAGGTGTGGAAAG	TGCCTTCTCTGTACAATCCTTCTCCTCTT
PtiMYB90	Potri.015G033600.1	GATGAAGATGACAGTGACGGAT	GATGAAGATGACAGTGACGGAT
PtiMYB121	Potri.002G185900.1	GAAGCTACGGAAAGGCCTGTGGTCAC	GCCGCAATCTGAGACCACCTGTTTC
PtiMYB125	Potri.003G114100.1	CTGCTGATCTTTTGCCAGAATC	GAATCCGAAATCGTGAACGC
PtiMYB128	Potri.003G132000.1	TGAGATGGATTAATTACTTGAGGCCTGATA	AAGGGAGCTGAAGACGAAAATAGGGTT
**Primers for gene clone**			
PtiCYP85A3	Potri.017G099000.1	ATGGCAGTTCTCTTGATGGTTCTTG	TTAGTGAGATGAGACCCTAATGTGTAGC
PtiMYB128	Potri.003G132000.1	ATGGGTCACCATTCTTGCTGCA	TTATAGAGATGAAGGGAAAGAAGAGAGTG
AtBZR1	AT1G75080	ATGACTTCGGATGGAGCTACGTCGACAT	TCAACCACGAGCCTTCCCATTTCC
**Primers for promoter clone**			
PtiCesA4	Potri.002G257900.1	CGGTTTTGGCCAGCTTTCAGTAC	TGAAGAGAAAGTGAAAGAAGGATGGTGAG
PtiCesA7-A	Potri.006G181900.1	CACCAGTGTTGAGCTTACCCAGACAGG	GACAAGTCCAGCACTGGCTTCCAT
PtiMYB128	Potri.003G132000.1	GTCAATCTGGAGGAAAAAAAAACAAATCAT	TTGATCCTTTCTTCCTCTTTTGTTTCTTTG

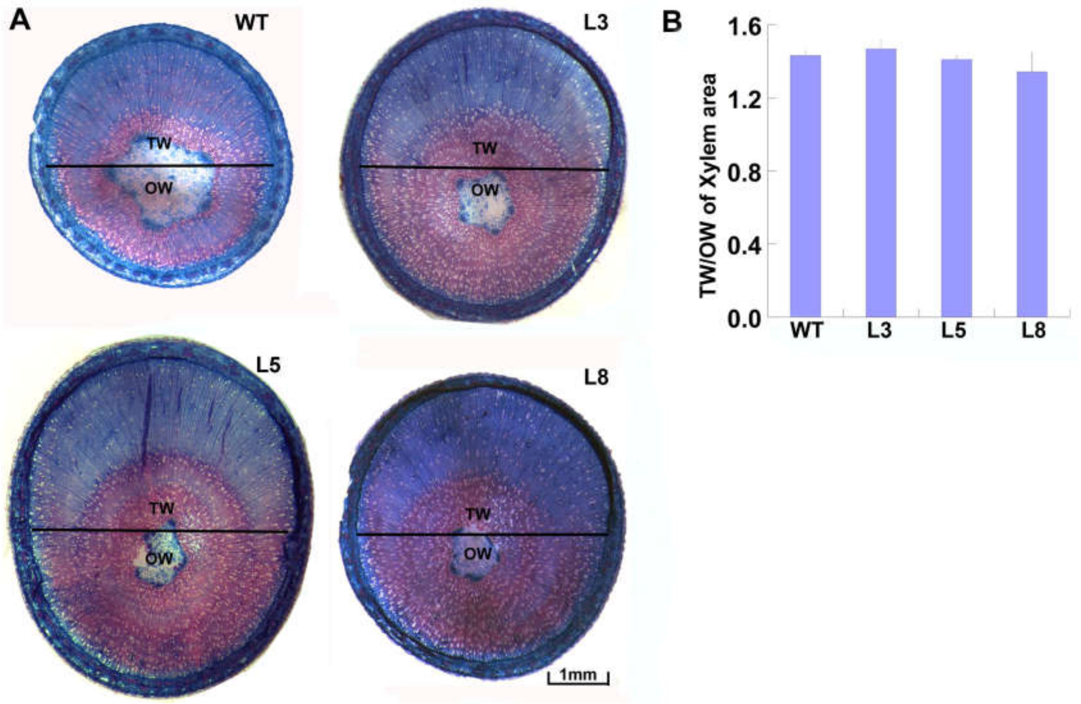

Figure S1. The area ratio of TW to OW in wild type (WT) and transgenic Shanxin yang plants overexpressing *PtiCYP85A3*. (A) Histochemical staining analyses. Cross sections taken from the inclined stems of WT and transgenic plants (L3, L5 and L8) were stained with safranin-O and astra-blue. TW xylem area was stained into blue. The horizontal lines indicate the division of TW and OW. TW, tension wood; OW, opposite wood. Scale bars = 1 mm. (B) TW to OW ratios in the xylem area of inclined stems. Data are means ± SD (n = 15) of three independent biological replicates.

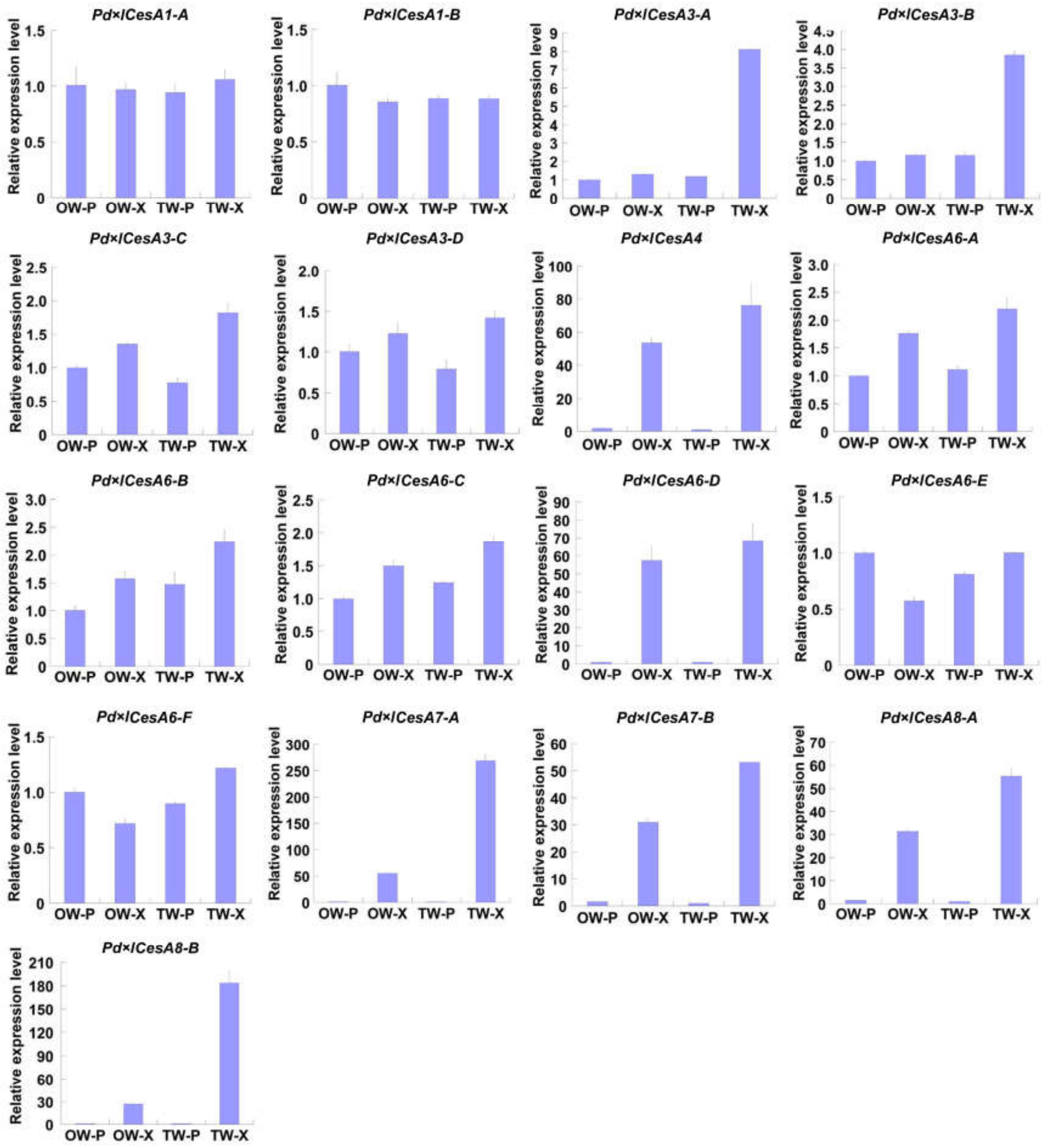

Figure S2. The expression patterns of *CesAs* during TW formation in wild typeShanxin yang plants. Expression of genes in the phloem tissue of OW was set to 1. Data are means ± SD (n = 6) of three independent biological replicates with three technical replicates each. OW-P, opposite wood phloem; TW-P, tension wood phloem; OW-X, opposite wood xylem; TW-X, tension wood xylem.

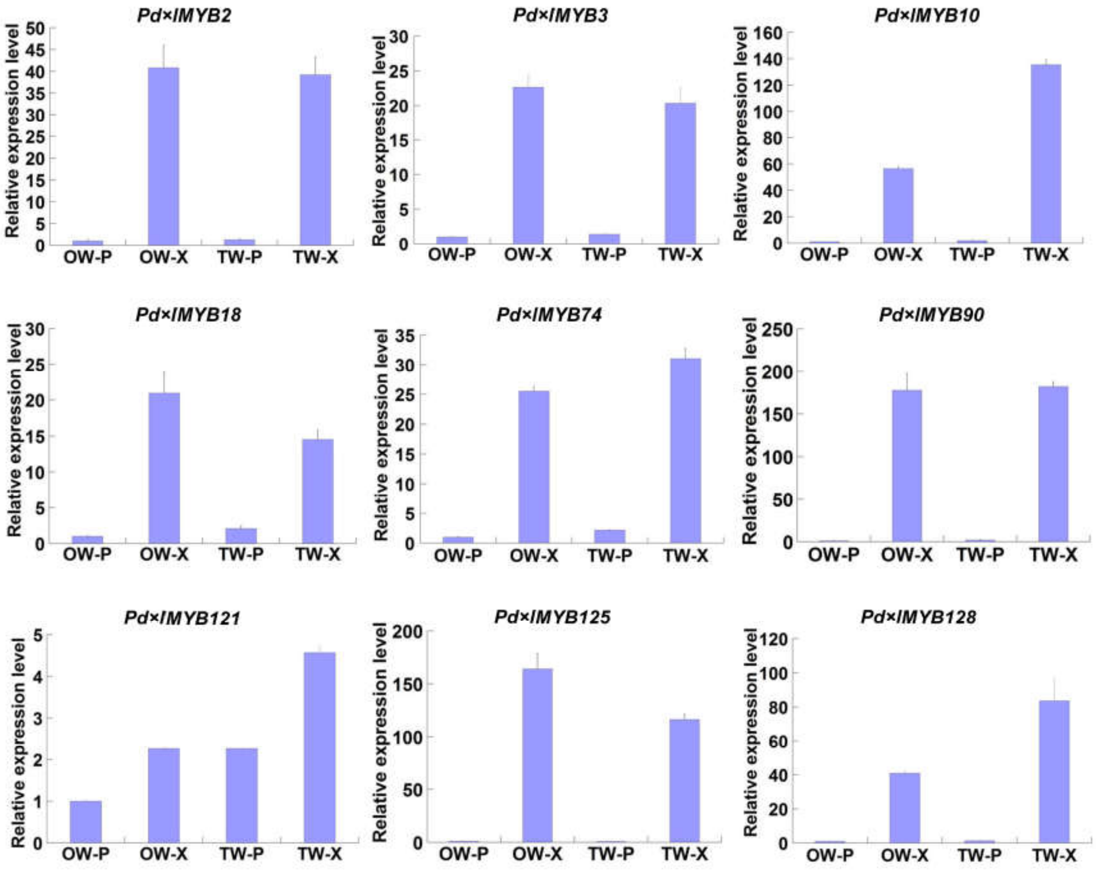

Figure S3. The expression patterns of xylem-related *MYBs* during TW formation in wild type Shanxin yang plants. Expression of genes in the phloem tissue of OW was set to 1. Data are means ± SD (n = 6) of three independent biological replicates with three technical replicates each. OW-P, opposite wood phloem; TW-P, tension wood phloem; OW-X, opposite wood xylem; TW-X, tension wood xylem.

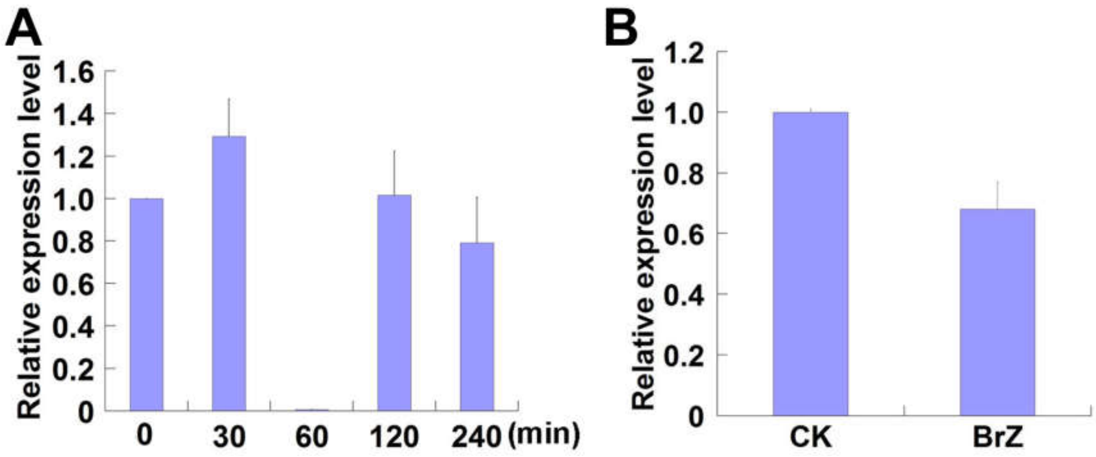

Figure S4. The relative expression of *MYB10* in wild type Shanxin yang plants treated with eBL and BrZ. (A) Expression analysis of *MYB10* in stem segments treated with 100 nM eBL for 0 min (as control), 30 min, 60 min and 240 min. (B) Expression of *MYB10* in TW xylem of stem segments treated with 5 mM BrZ for ten days. Stem segments treated without BrZ was used as control. Data are means ± SD (n = 6) of three independent biological replicates with three technical replicates each.

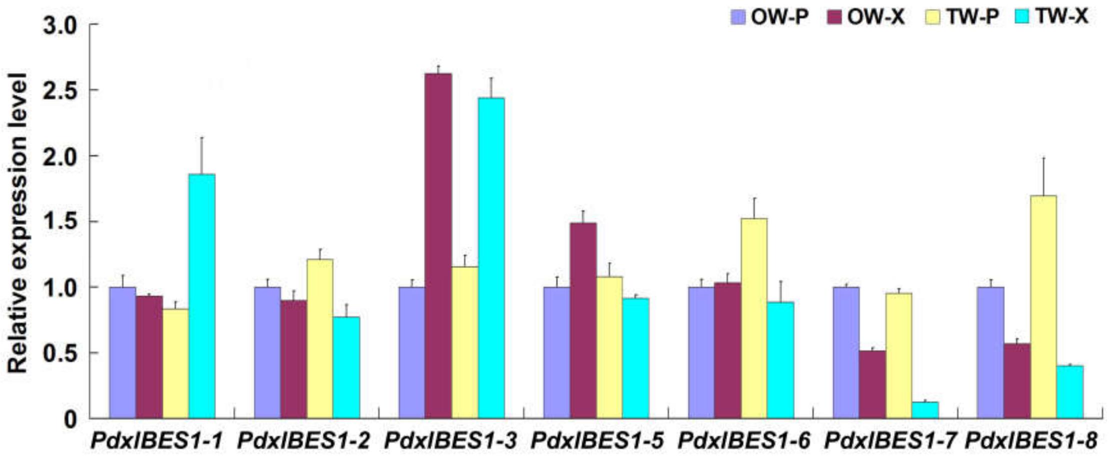

Figure S5. The expression patterns of putative poplar *BESs* during TW formation in wild type Shanxin yang plants. Based on the sequence of AtBZR1, seven putative *BZR1*/*BES1* genes were found from poplar genome and named BES1-1 to BES1-8. Expression of genes in the phloem tissue of OW was set to 1. Data are means ± SD (n = 6) of three independent biological replicates with three technical replicates each. OW-P, opposite wood phloem; TW-P, tension wood phloem; OW-X, opposite wood xylem; TW-X, tension wood xylem.

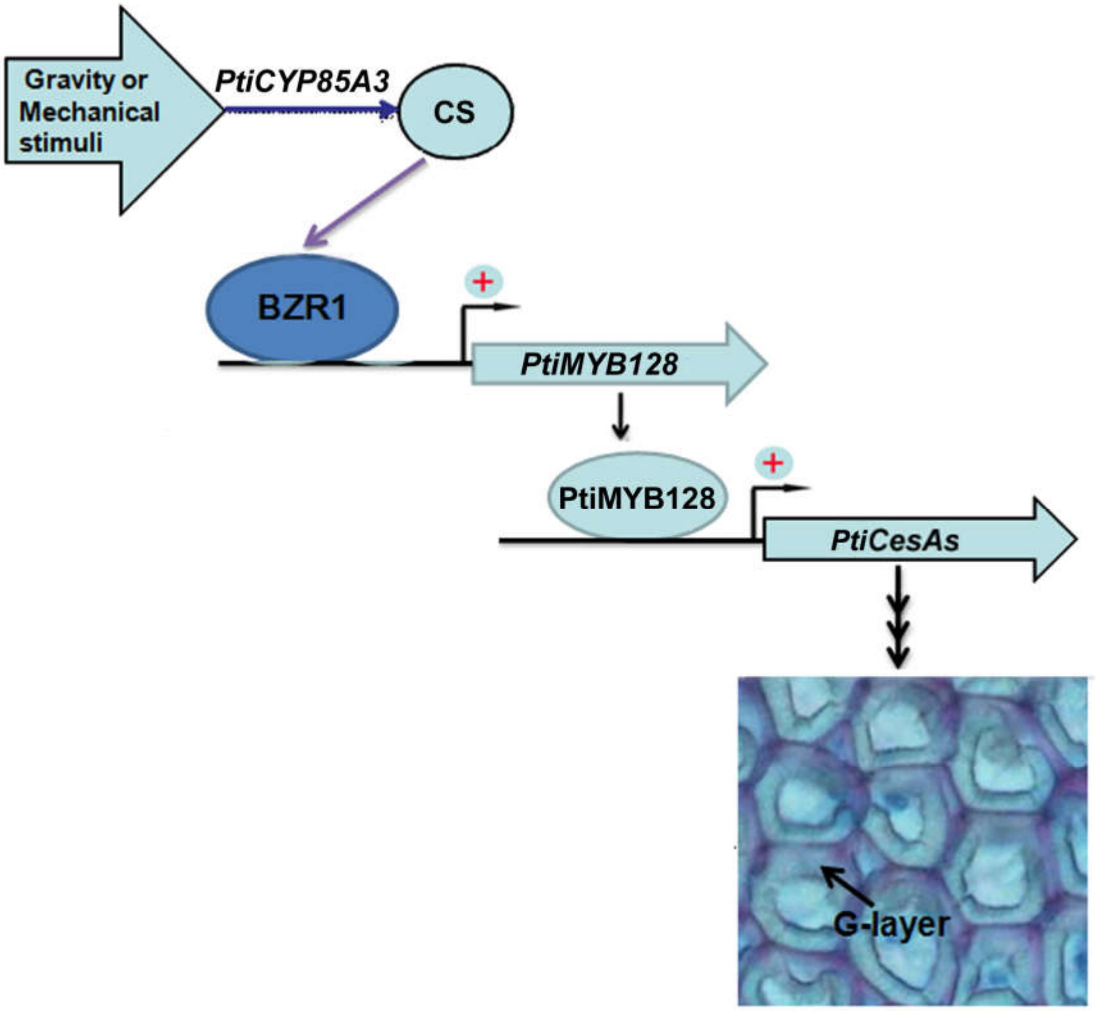

Figure S6. A proposed model of BR regulated G-layer formation in poplar. When poplar plants were stimulated with external force or gravity, the expression of *PtiCYB85A3* was up-regulated to promote the production of CS in the TW xylem. Then expression of BZR1 in the TW xylem was activated to activate the expression of TW-associated transcription factor PtiMYB128, which up-regulated the transcription of TW-associated *PtiCesAs*, leading to enhanced cellulose biosynthesis and G-layer thickness in the TW xylem fiber cells.
